# Inferring neurocognition using artificial intelligence on brain MRIs

**DOI:** 10.3389/fnimg.2024.1455436

**Published:** 2024-11-27

**Authors:** Mohammad Arafat Hussain, Patricia Ellen Grant, Yangming Ou

**Affiliations:** ^1^Department of Pediatrics, Boston Children's Hospital, Harvard Medical School, Boston, MA, United States; ^2^Department of Radiology, Harvard Medical School, Boston, MA, United States; ^3^Computational Health Informatics Program, Boston Children's Hospital, Harvard Medical School, Boston, MA, United States

**Keywords:** neurocognition, artificial intelligence, brain MRI, intelligence, P-FIT model

## Abstract

Brain magnetic resonance imaging (MRI) offers a unique lens to study neuroanatomic support of human neurocognition. A core mystery is the MRI explanation of individual differences in neurocognition and its manifestation in intelligence. The past four decades have seen great advancement in studying this century-long mystery, but the sample size and population-level studies limit the explanation at the individual level. The recent rise of big data and artificial intelligence offers novel opportunities. Yet, data sources, harmonization, study design, and interpretation must be carefully considered. This review aims to summarize past work, discuss rising opportunities and challenges, and facilitate further investigations on artificial intelligence inferring human neurocognition.

## 1 Introduction

Neurocognition refers to the *mental process* of learning, solving problems, remembering, and appropriately using information from memory (Morley et al., 2015). On the other hand, intelligence refers to different *mental abilities* such as problem-solving, logic, reasoning, and planning. Intelligence describes *neurocognition quality* in people (Latal et al., 2016; Kessler et al., 2020; Watson et al., 2018). A person's neurocognition and its manifestation in terms of intelligence are important factors in a person's education, career, social status, health, and longevity (Dubois et al., 2018b). Yet, how can we explain the substantial differences among people in their neurocognition? Can we effectively measure a person's neurocognition? Can we predict a person's future course of neurocognition, in normal and disease? Seeking answers to these questions has been at the core of neuroscience research for over a century. The hope is to identify and boost each individual's potential (different people are “smart” in different ways) (Kanai and Rees, 2011) and to intervene early and improve outcomes for those vulnerable (Liamlahi and Latal, 2019; Urschel et al., 2018).

Differences in neuroanatomy and brain connectivity are widely believed to contribute to individual variability of neurocognition (Kanai and Rees, 2011). Early studies (the 1900s) related neurocognitive functions to brain structures in post-mortem brains (Spitzka, 1903). The invention of magnetic resonance imaging (MRI) in 1977 has allowed for the *in vivo*, three-dimensional (3D) study of brain structure and function. Advancement in MRI analytics in the past four decades further brings the automated, quantitative, and sophisticated investigation of neuroanatomy (Pol et al., 2006; Rushton and Ankney, 2009), white matter integrity (Deary et al., 2006; Schmithorst et al., 2005), and brain circuit connectivity (Jensen, 2006), which are all found to correlate to neurocognitive and intelligence test scores (Kanai and Rees, 2011). Sample sizes, however, were often dozens to lower hundreds; findings were not always consistent; and population-level associations have not yet been reliably translated into individual prediction.

The very recent availability of big data brain MRI (over 1,000 or even 10,000 individuals) (Poldrack and Gorgolewski, 2014), coupled with the rise of artificial intelligence (AI) (Graham et al., 2020), promises to revolutionize MRI inference of neurocognition. While opportunities arise, open issues on the data source, merging, harmonization, analytics, target test scores, study design, and interpretations must be considered. As mentioned earlier, human intelligence reflects the quality of neurocognition functions (Latal et al., 2016; Kessler et al., 2020; Watson et al., 2018); recent reviews mostly focused on MRI's association with human intelligence (Dizaji et al., 2021). In contrast, this review focuses on the association of MRI with neurocognition/intelligence at the population level and the prediction of individual neurocognition/intelligence. We conducted a comprehensive analysis of studies on the association of population-level neurocognition with brain MRI and predicting an individual's neurocognition/intelligence from brain MRI using predictive models, leveraging Google Scholar for a thorough review of the most relevant literature. We also discuss open issues and rising opportunities. The aim is to facilitate further studies of artificial intelligence inferring human intelligence.

### 1.1 Search strategy

We searched Google Scholar thoroughly for all scholarly publications: peer-reviewed journal papers and papers published in the proceedings of conferences or workshops from January 2005 to August 2024. Our search query was (Magnetic Resonance^*^ | MRI^*^) (Cognition | Neurocognition | IQ | Intelligence) (Correlat^*^ | Predict^*^). We applied a rigorous selection process to identify relevant articles for our review. The criteria for inclusion were: (1) the full text had to be accessible online or published in reputable journals or conferences indexed in databases such as PubMed, IEEE Xplore, Scopus, or Web of Science; (2) the article must have utilized traditional statistical or conventional machine learning or deep learning, specifically for finding correlation of neurocognition with different brain MRIs or for the prediction of neurocognition/intelligence from different brain MRIs; (3) the hypothesis posed by the study had to be supported by robust qualitative and quantitative results; and (4) the article had to meet a minimum quality standard, ensuring no missing abstracts or methodologies, no reference errors, and clear figure legends and axis titles. Similar search strategies and selection criteria have been used in other recent reviews (e.g., Azad et al., 2024). In addition, we took great care to include all relevant studies utilizing different MRI modalities and AI for neurocognition prediction, though a few papers may have been inadvertently overlooked. Our goal, however, was to provide a comprehensive overview of the field. In total, we have reviewed 94 articles in this study.

## 2 Measurement of human neurocognition

Assessment of human neurocognitive abilities is often performed via the assessment of human intelligence (Latal et al., 2016; Kessler et al., 2020; Watson et al., 2018). Intelligence is positively correlated to different neurocognitive abilities such as processing speed (Watson et al., 2018), executive functions (Naef et al., 2021; Fontes et al., 2019), general memory (Pike et al., 2021), and working memory (Ehrler et al., 2020). That is why, the estimation of human intelligence (see Sections 2.1–2.3 for details) lies at the core of assessing different neurocognitive abilities. As such, this review also includes studies that link human intelligence scores with brain MRIs.

### 2.1 Cattell–Horn–Carroll's theory for human intelligence

**Figure 1:**
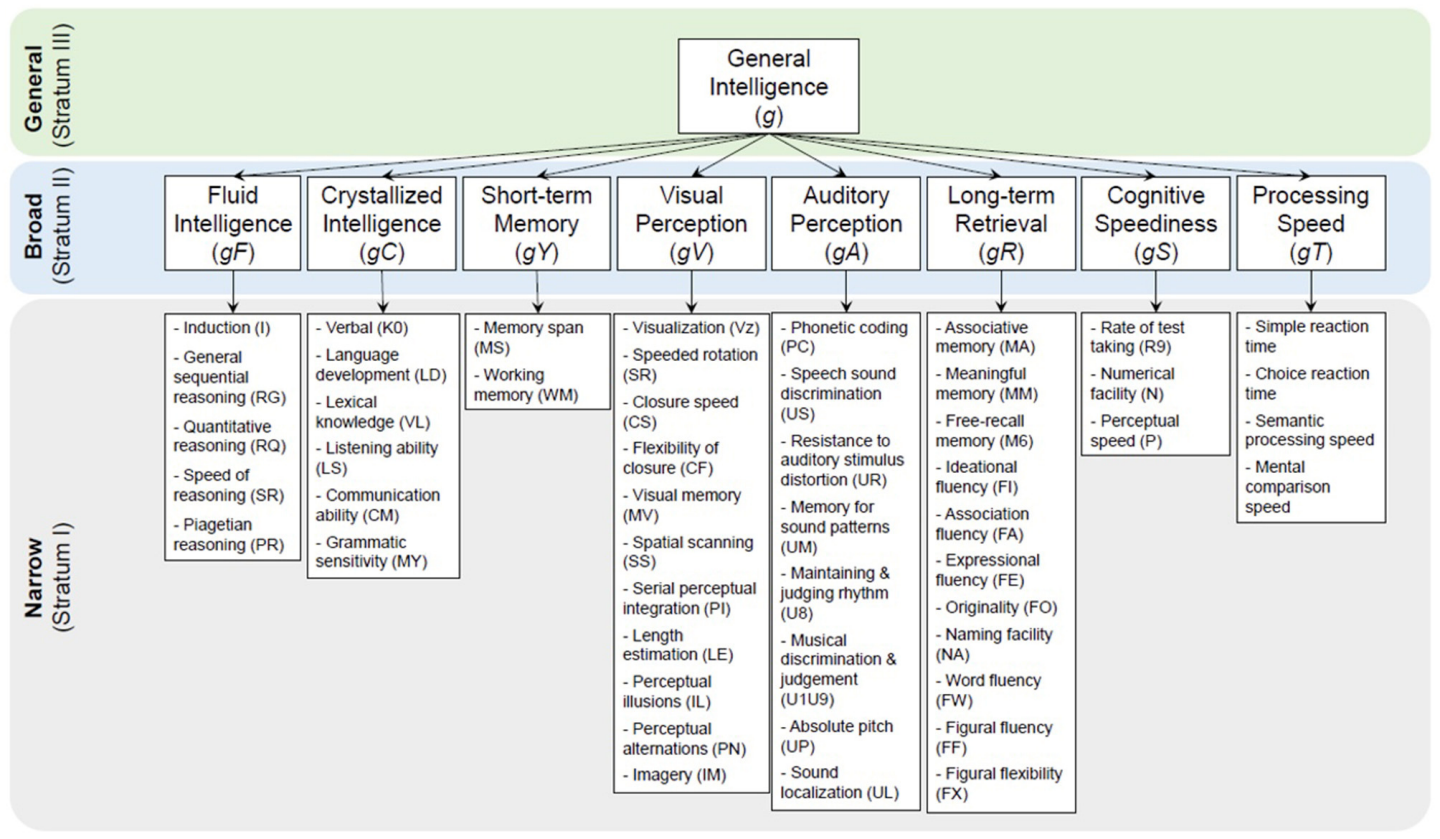
The Cattell-Horn-Carrol (CHC) Theory divides the general intelligence *g* (top stratum), which is hard to measure, into 8–10 broad abilities (middle stratum; different key neurocognitive functions) and over 60 narrow abilities (bottom stratum), which are more measurable. Measuring these narrow abilities is the core of many of today's neurocognitive and intelligence tests.

The Cattell-Horn-Carroll (CHC) theory (McGrew, 2009) is a widely accepted framework for intelligence tests. It categorizes intelligence into three strata (Carroll, 1993; Horn and Cattell, 1966): general intelligence (*g*) (Spearmen, 1904), broad abilities (Cattell, 1963), and narrow abilities (Figure 1). The *g*, proposed by Spearmen (1904), is a fundamental ability supporting all neurocognitive abilities. Broad abilities include factors like fluid (*gF*) and crystallized (*gC*) intelligence, short-term memory (*gY*), long-term retrieval (*gR*), visual perception (*gV*), auditory perception (*gU*), cognitive speediness (*gS*), and processing speed (*gT*). Each broad ability is further divided into narrow abilities.

### 2.2 Intelligence and neurocognition tests

The CHC theory simplifies the measurement of *g* by testing 8-10 broad abilities and over 60 narrow abilities (McGrew, 2009; Kaufman, 2018). Tests like the Wechsler Adult Intelligence Scale IV (WAIS-IV) (Hartman, 2009) assess five broad abilities to estimate the full-scale intelligence quotient (FSIQ) as a proxy of *g* (Benson et al., 2010). Other popular scoring systems like the Wechsler Intelligence Scale for Children-V (WISC-V), Wechsler Abbreviated Scale of Intelligence-II (WASI-II) (Wechsler, 1999), and others follow similar sub-factoring for IQ scoring.

### 2.3 Cognitive test batteries

FSIQ or *g*, while indicative of overall cognitive ability, does not express the extent of impairment in single domains (Kubinger, 2019). To examine specific broad abilities, cognitive test batteries are used. These tests assess performance in several domains, including additional ones like executive function and language performance. For instance, the neuropsychological assessment battery (NAB) assesses five cognitive domains. Another popular and widely used test battery, the NIH toolbox of neurocognitive battery (NIH-TCB) (Akshoomoff et al., 2013) is designed to measure (i) executive function, (ii) attention, (iii) episodic memory, (iv) language, (v) processing speed, and (vi) working memory (Denboer et al., 2014).

## 3 Theories linking brain structure and neurocognitive functions

Neuroimaging studies since the 1980s have given rise to theories about brain structure-function mapping. Examples include network neuroscience theory (NNT) (Barbey, 2018), lateral prefrontal cortex theory (LPFCT) (Duncan and Owen, 2000), multiple-demand theory (M-DT) (Duncan, 2010), and process overlap theory (POT) (Kovacs and Conway, 2016). Among these popular theories is the Parieto-Frontal Integration Theory (P-FIT) (Jung and Haier, 2007). The P-FIT theory, as detailed in Section 3.1, is influential as it offers insights that human intelligence/neurocognition resides in large-scale connected brain regions known as brain networks (Deary et al., 2010).

The selection of the P-FIT as a central framework in this study, where findings in the reviewed manuscripts in this study are juxtaposed, is grounded in its unique integration of structural and functional neuroimaging findings across multiple studies. While other theories, including NNT, LPFCT, and M-DT, offer valuable insights into brain structure-function relationships, P-FIT stands out for its comprehensive scope. It synthesizes evidence from various neuroimaging modalities, such as structural, diffusion, and functional MRIs, to link specific brain regions and networks with intelligence. A critical strength of P-FIT is that it consolidates findings across 37 independent neuroimaging studies, as originally outlined by Jung and Haier (2007), which focused on brain regions like the parietal and frontal cortices that have repeatedly been implicated in neurocognitive processing. Furthermore, this theory focuses on large-scale brain networks and is supported by an increasing number of studies demonstrating network integrity's importance in sustaining human intelligence. Recent evidence consistently aligns with the central claim of the P-FIT that interconnected regions across the brain, rather than isolated structures, underlie complex cognitive functions. In contrast, other models like M-DT and LPFCT either lack the same breadth of empirical validation or focus more narrowly on task-specific activations, which do not capture the full spectrum of intelligence-related processes. Therefore, the P-FIT is perhaps the most studied theory that emphasizes network integrity in the sustenance of human intelligence (Dizaji et al., 2021) based on its robust empirical support and broad explanatory power, as reflected in the current neuroimaging literature.

### 3.1 The P-FIT theory for distributed brain network underlying human intelligence

**Figure 2:**
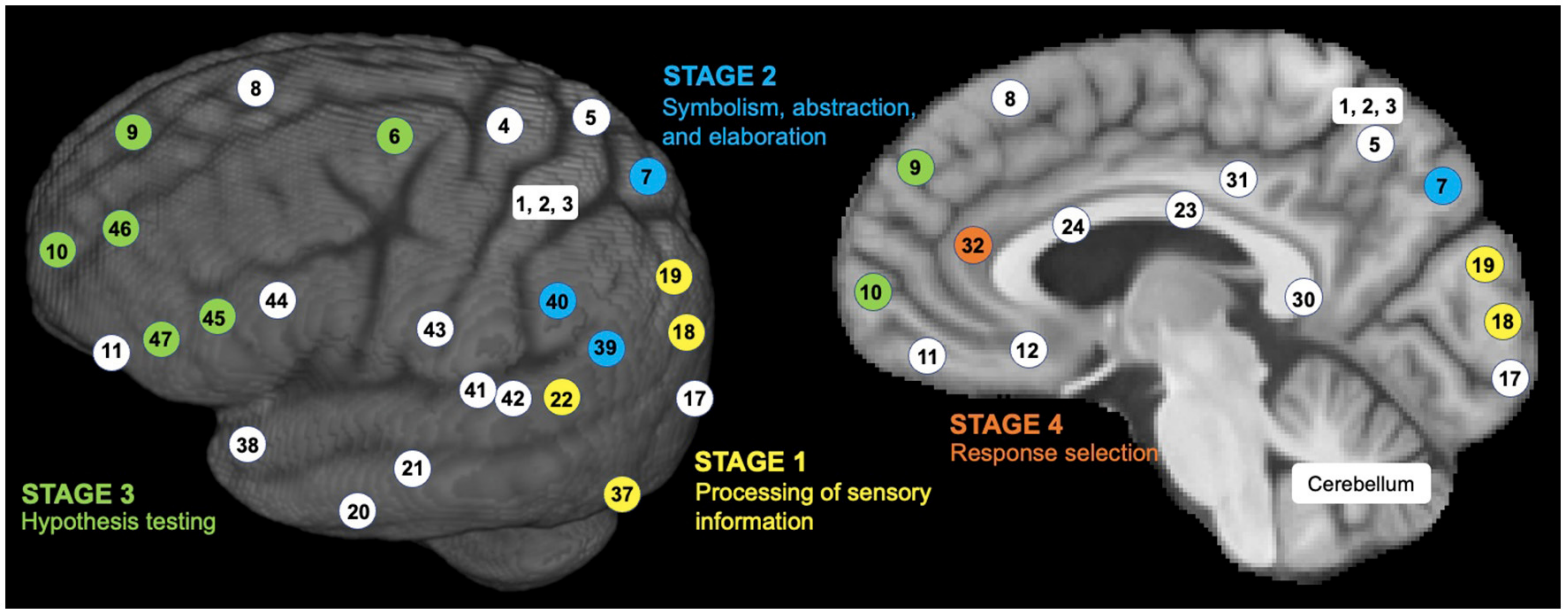
Sensory information processing stages by the P-FIT model. Brodmann area (BA) numbers are color-coded to correspond to different stages of information processing (Stage 1 as yellow, Stage 2 as blue, Stage 3 as green, and Stage 4 as orange; a few white-colored BAs are shown for reference).

The P-FIT theory emphasizes network integrity most in the sustenance of human intelligence (Dizaji et al., 2021). The P-FIT theory involves four information processing stages, each involving different Brodmann areas (BAs) in the connected brain networks (Jung and Haier, 2007; Colom et al., 2022) (see Figure 2):

**Stage 1:** It is assumed that humans first gather and process sensory information predominantly in the occipital and temporal areas (i.e., brain regions colored with yellow numbers in Figure 2). Early processing of sensory information happens in the extrastriate cortex (BAs 18 and 19). Recognition, imagery, and elaboration happen in the fusiform gyrus (BA 37). Analysis and elaboration of auditory information syntax happen in Wernicke's area (BA 22).

**Stage 2:** This stage involves the structural symbolism, abstraction, and elaboration of the basic sensory information (in Stage 1) in the angular gyrus (BA 39), supramarginal gyrus (BA 40), and superior parietal lobule (BA 7). These brain regions are colored by blue numbers in Figure 2.

**Stage 3:** This stage involves the interaction between parietal areas and frontal lobes (BAs 6, 9, 10, 45, 46, and 47, as colored by green numbers in Figure 2). This interaction supports problem-solving, evaluation, and hypothesis testing.

**Stage 4:** Once the best solution is reached, the anterior cingulate (BA 32) gets engaged for response selection and inhibition of competing responses. This brain region is colored by an orange number in Figure 2.

The P-FIT theory emphasizes that the whole process (Stages 1–4) depends upon the fidelity of underlying white matter connectivity. White matter facilitates rapid and error-free data transmission from the posterior to frontal brain regions. Note that the P-FIT model considers only those Brodmann areas, which appeared in more than 25% of the total 37 studies (Jung and Haier, 2007) reviewed. Table 1 covers a full spectrum of Brodmann areas that Jung and Haier (2007) have summarized.

**Table 1:** A list of Brodmann areas (BAs) that were found to be related to human cognition and intelligence in a total of 37 studies over 1,557 subjects (Jung and Haier, 2007).

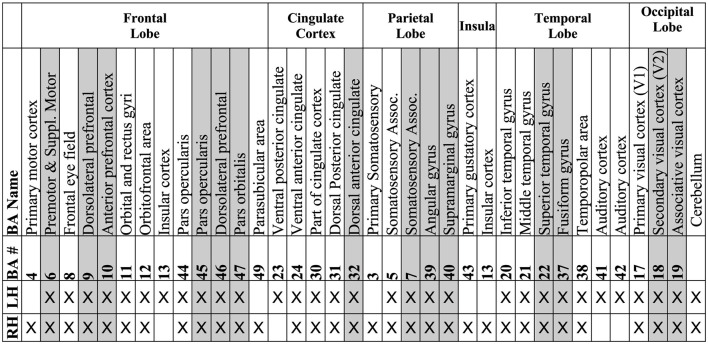

Only shaded columns of BAs comprise the P-FIT model. BA, brodmann area; LH, left hemisphere; RH, right hemisphere.

## 4 Methods inferring neurocognition from brain MRIs

**Figure 3:**
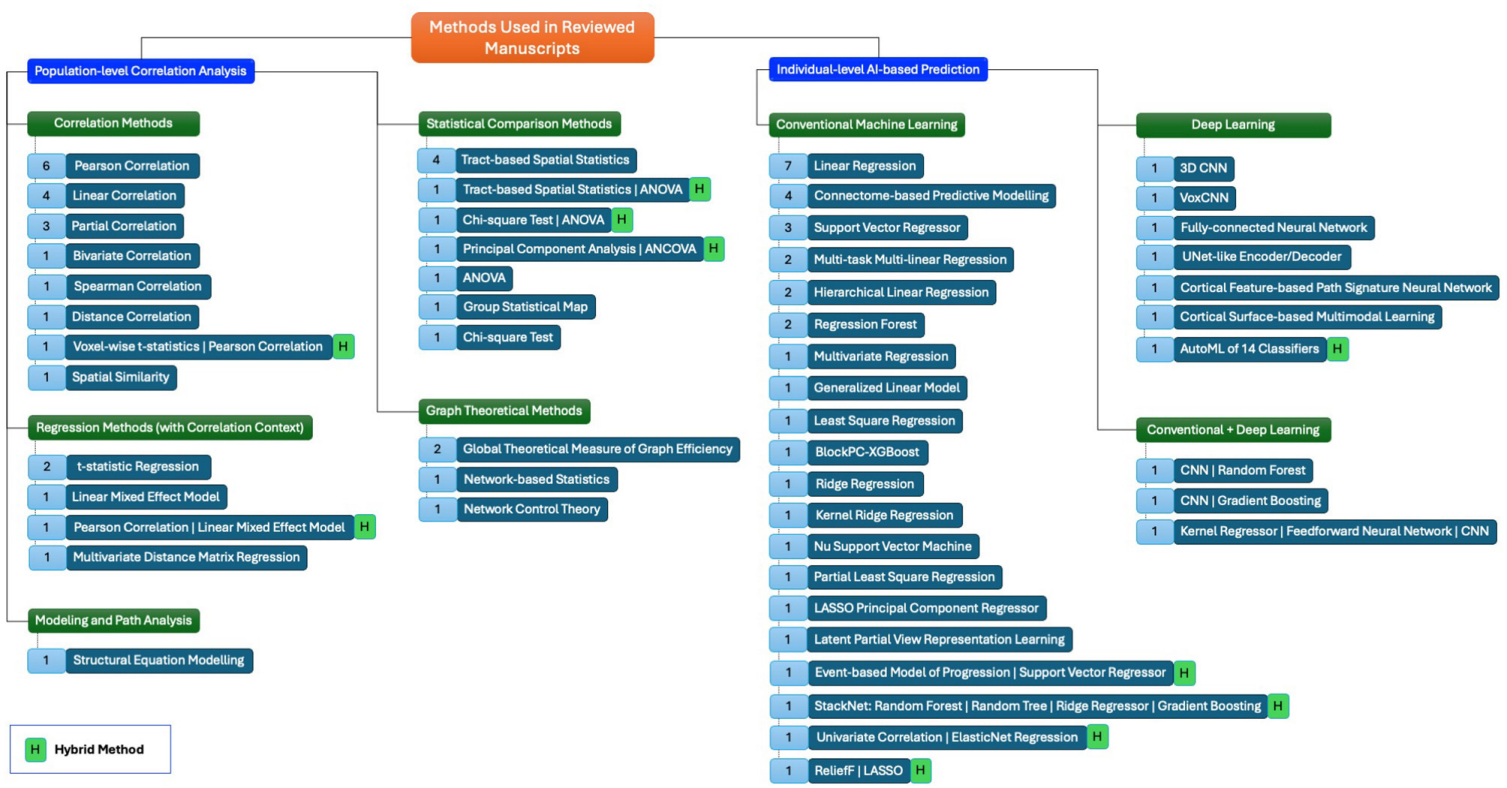
A mind map showing the methods, and their types. Numbers in blue boxes appended in the front of each method represent the number of papers used in that method.

In our review, the application of traditional methods for correlating neurocognitive outcomes with brain MRI data or AI methods for predicting neurocognitive outcomes from brain MRI data is categorized into several broad methodological approaches, as outlined in Figure 3. These include both traditional/statistical and AI-based methods. We distinguish between population-level correlation analysis methods and individual-level prediction methods, each with distinct advantages and limitations. Below, we briefly describe these categories, their relative advantages and disadvantages, and how AI benefits brain analysis beyond traditional approaches.

### 4.1 Population-level correlation analysis methods

As shown in Figure 3, traditional approaches like correlation methods (e.g., Pearson and Spearman correlations) and regression methods (e.g., linear regression and mixed-effect models) are frequently used to assess relationships between brain imaging features and neurocognitive measures at the population level. These methods are widely employed due to their simplicity and interpretability, making them useful for understanding general trends across populations.

However, these traditional methods often struggle with the high dimensionality of neuroimaging data. Figure 3 also highlights statistical comparison methods (e.g., ANOVA, chi-square) that are used for population-level comparisons, though they provide limited insight into correlations between two types of parameters. More advanced techniques such as graph theoretical methods (e.g., global efficiency and network-based statistics) and modeling and path analysis methods (e.g., structural equation modeling) offer deeper insights into brain network properties and complex interactions but still lack the predictive power of AI-based approaches.

### 4.2 Individual-level prediction methods

Figure 3 further outlines AI approaches designed for individual-level neurocognition prediction, which aimed a superior performance in identifying relationships between brain MRI features and neurocognitive outcomes at the individual level, a task where traditional methods fall short. These approaches are divided into three broad categories.

#### 4.2.1 Conventional machine learning

Methods such as support vector machines (SVM), random forests, and connectome-based predictive modeling (CPM) have been extensively used to predict neurocognitive outcomes from MRI data. These models excel at handling complex, multivariate datasets and can identify patterns that simpler models cannot. However, they require manual feature selection, which can introduce bias and limit generalizability across different datasets.

#### 4.2.2 Deep learning

Deep learning models, such as convolutional neural networks (CNNs) and their different variations (e.g., 3D CNN, VoxCNN, UNet, etc.), have recently gained traction for brain MRI analysis. These models automatically learn features from raw data, allowing for the detection of non-linear relationships and subtle patterns in brain structure and function that are crucial for accurate neurocognitive predictions. While deep learning models offer superior performance, their lack of interpretability and need for large datasets are notable challenges.

#### 4.2.3 Combination of conventional machine learning and deep learning

In some studies, approaches that combine conventional machine learning with deep learning have emerged (e.g., CNN & Random Forest, CNN & Gradient Boosting, etc.). These methods aim to leverage the interpretability of conventional models with the powerful feature extraction capabilities of deep learning. For instance, conventional machine learning may be used for feature selection, followed by deep learning for final prediction, offering improved prediction accuracy while retaining a degree of interpretability.

### 4.3 Advantages of AI over traditional methods

AI approaches, particularly conventional machine learning, deep learning, and a combination of them, aimed to offer distinct advantages over traditional population-level correlation analysis methods. AI models are highly effective at managing the high dimensionality and complexity of brain MRI data, whereas traditional methods, such as correlation and regression, are limited in their capacity to generalize to new data or make individual-level predictions.

AI techniques also enable the modeling of non-linear relationships between brain imaging data and neurocognitive outcomes, which traditional linear methods often miss. The individual-level predictions facilitated by AI are particularly valuable for precision medicine and monitoring cognitive decline, where traditional population-level analyses fail to capture the nuances of individual variability.

Despite these strengths, AI approaches, particularly deep learning models, are often criticized for their lack of interpretability, an issue that is less of a concern with traditional/statistical methods, which are more transparent in their underlying assumptions and relationships. However, the ability of AI to capture complex and subtle patterns gives it a significant advantage in predicting brain-behavior relationships.

## 5 Structural MRI to infer neurocognition

Typical brain MRIs include structural, diffusion, and functional sequences. This section starts with structural MRI (sMRI) and its inference of human intelligence and neurocognition.

sMRI sequences typically include T1- and T2-weighted MRI (T1/T2-MRI). Subsections below will introduce morphometric features from sMRI [see review for more details (Lerch et al., 2017)] and their use to infer neurocognition and intelligence.

### 5.1 Total and regional brain volume to infer neurocognition

Several studies have investigated the relationship between brain volume, both total and regional, and neurocognitive outcomes using population-level statistical approaches. Software tools such as FreeSurfer (Fischl, 2012), FSL (Jenkinson et al., 2012), AFNI (Cox, 2012), and others [see review (Eickhoff et al., 2018)] allow for the segmentation of T1- or T2-weighted MRIs into hemispheres, tissue types (e.g., white matter, gray matter, cerebrospinal fluid), and brain regions using single/multi-atlas (Zhang-James et al., 2019) or machine learning (Chen et al., 2018) techniques (see Supplementary Figure 1). However, it is important to acknowledge that findings in the literature regarding brain volumes and cognitive functions may vary across studies, and not all results have been widely replicated or universally confirmed. Some conclusions are based on specific cohorts or methodologies, which could lead to variability in outcomes.

#### 5.1.1 Population-level correlation analysis

Nave et al. (2019) examined the population-level correlation of total brain volume (TBV), the combined volume of gray matter, white matter, and cerebrospinal fluid with fluid intelligence (*gF*) in a large cohort of adults (*N* = 13,608), reporting a modest positive correlation (*r* = 0.19, *p* < 0.05). However, this finding reflects a population-level trend and does not necessarily translate to predictive power at the individual level. Individual-level inference of neurocognition from TBV is notably sparse in the literature, highlighting the uncertainty in applying these correlations to personalized predictions. Similarly, other studies reported positive correlations between total gray matter volume and cognitive functions such as fluid intelligence (*r* = 0.16, *p* < 0.01), working memory (*r* = 0.21, *p* < 0.01), and quantitative reasoning (*r* = 0.26, *p* < 0.01) in a smaller adult cohort (*N* = 211) (Paul et al., 2016). These results offer valuable insights, but the field could benefit from further replication efforts to confirm the robustness of these findings across diverse populations and study designs, as this study data is not publicly accessible, and the sample size is small. In an infant cohort, studies reported that pre-term fetal growth-restricted (PT-FGR) infants had lower gray matter, white matter, and other brain region volumes compared to pre-term appropriate gestational age (PT-AGA) and term AGA groups (Morsing et al., 2018). This reduction in brain volumes corresponded to lower FSIQ scores in the PT-FGR group compared to PT-AGA (80 vs. 103, respectively). However, it is worth noting that not all studies on early brain development and cognition reach identical conclusions, and differences in study design (e.g., imaging techniques, timing of assessment) may account for some of the variability observed in the literature. Furthermore, prediction of insight test battery (ITB) cognitive scores from gray matter volumes in regions such as the right insula and right middle cingulate cortex/precuneus (BAs 13, 14, 16, 4) has shown significant results (*p* < 0.001) (Ogawa et al., 2018), further underscoring the role of regional volumes in neurocognitive assessments.

#### 5.1.2 Individual-level AI-based predictions

AI-based methods have emerged to make individualized predictions of neurocognitive performance by utilizing more detailed brain features, such as regional volumes, rather than relying solely on TBV. Studies using machine learning (Zhang-James et al., 2019; Chiang et al., 2019; Srivastava et al., 2019; Ren et al., 2019; Tamez-Pena et al., 2019; Brueggeman et al., 2019; Mihalik et al., 2019; Ranjbar et al., 2019; Wlaszczyk et al., 2019; Kao et al., 2019; Li et al., 2019; Saha et al., 2021) have explored regional volumes, identifying key brain regions, such as the frontoparietal (BAs 6, 8, 9), cingulo-opercular (includes BAs 22, 41, and 42), visual (includes BAs 17, 18, and 19), somatosensory (includes BAs 1, 2, 3, 5, and 7), right posterior cingulate gyrus (BAs 23, 31), entorhinal white matter (BA 28), globus pallidus, precentral gyrus (BA 4), corpus callosum, left/right hippocampus, parahippocampal gyrus (BA 34), thalamus, precentral gyrus (BA 4), caudate nucleus, pons, and motor (includes BAs 4 and 6) cortex areas, as significant predictors of residual fluid intelligence (*gF*) in adolescents. These models reported mean squared errors (MSEs) ranging from 92 to 101 for a range of true residual fluid intelligence scores of [−40, 30], indicating moderately accurate predictions at the individual level. However, the predictive power of these models can vary; for instance, some studies reported correlations as low as *r* = 0.1 (*p* < 0.05) between predicted and actual fluid intelligence (*gF*) scores (Saha et al., 2021). This suggests that while AI-based methods offer promise, there remains uncertainty regarding their generalizability across methods. Other studies have extended these predictions to FSIQ (or *g*) using models that integrate brain volumes across networks such as the frontoparietal network (BAs 6, 8, 9), default mode network (BAs 38, 25, 23, 31, 4), dorsal attention network (BAs 17, 18, 19, 8, 7, 6), and cerebellum. These approaches used principal component analysis (PCA) to reduce dimensionality and subsequently used linear support vector regressor on the PCA features. They reported MSEs of around 320 (*p* = 0.279) and correlations of *r* = 0.11 between predicted and actual FSIQ scores for true residual FSIQ in the range of (Jung and Haier, 2007; Hilger et al., 2020; Santarnecchi et al., 2017). In addition, caudate nucleus volumes have been found to play an important role in individual cognitive predictions, particularly in reinforcement learning and decision-making processes (Packard and Knowlton, 2002; Tricomi et al., 2006). A significant positive correlation (*r* = 0.24, *p* = 0.01) between caudate volume and FSIQ has been reported (Grazioplene et al., 2015), reinforcing its importance in neurocognitive assessments. While AI-based approaches hold significant promise for individual-level predictions using local and total brain volumes, the variability in their accuracy highlights the need for further research and validation across larger and more diverse cohorts.

#### 5.1.3 Salient brain regions across various neurocognitive measures

**Figure 4:**
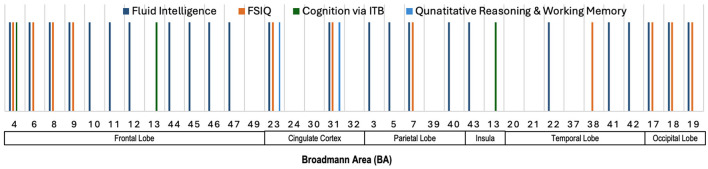
Illustration showing the Broadmann Areas, which have been found salient at least once in one or more reviewed articles, reporting an association of total brain volume or regional brain volume with various neurocognitive measures.

Figure 4 provides an illustration highlighting the Broadmann Areas (BAs) that have been identified as salient in at least one of the reviewed studies investigating associations between total or regional brain volume and various neurocognitive measures. This figure serves to visually summarize the brain regions that were most frequently reported as having significant correlations with neurocognitive outcomes, thereby offering an integrative perspective on the structural correlates of cognition. We see in this figure that multiple BAs within the frontal lobe, such as BAs 4, 6, 8, and 9, were consistently implicated in studies examining fluid intelligence and FSIQ. These findings highlight the importance of the frontal lobe in supporting a wide range of cognitive functions. Similarly, BAs 23 and 31 in the cingulate cortex were linked to fluid intelligence, FSIQ, and Quantitative Reasoning & Working Memory. Furthermore, BA 7 in the parietal lobe, and BAs 17, 18, and 19 in the occipital lobe were primarily linked to fluid intelligence and FSIQ, highlighting their importance for general cognitive abilities.

### 5.2 Cortical surface metrics to infer neurocognition

In addition to the total and regional brain volumes, metrics on the cortical surface (e.g., thickness, area, curvature, gyrification, etc.) also play a vital role in inferring human intelligence. Automated software such as FreeSurfer can reconstruct brain cortical surfaces and extract cortical surface areas, cortical thickness, cortical folding curvatures, and gyrification indices (see Supplementary Figure 1). In this section, we discuss those studies that included cortical surface metrics with or without cortical volumes (summarized in Supplementary Table 2).

#### 5.2.1 Population-level correlation analysis

Previous research suggests that information integration and processing are supported by regions such as the parahippocampal gyrus (BA 34) and the precuneus/cuneus cortex (BAs 4, 19) (Pol et al., 2006; Westlye et al., 2009), while the ventral temporal cortex (Bar et al., 2001; McCandliss et al., 2003) is implicated in visual identification and recognition. Additionally, the integration and retrieval of semantic knowledge are associated with the medial temporal lobes (BA 38) (McClelland and Rogers, 2003). However, these functions likely emerge from the coordinated activity of multiple brain regions rather than being confined to isolated areas. Cortical surface metrics are often combined with regional cortical volumes as features to enhance the prediction of neurocognitive outcomes. For instance, significant positive correlations were found between the Reynolds Intellectual Assessment Scales (RIAS) composite IQ scores and cortical surface area, cortical thickness, and gray matter volumes in the orbitofrontal gyrus (BAs 11, 12) (*r* = 0.41; *p* = 0.03) and transverse temporal gyri (BAs 41, 42) (*r* = 0.42; *p* = 0.02) (Li et al., 2020). Similar relationships were reported for the left superior temporal gyrus (BA 22) (*r* = 0.41; *p* = 0.04) and right anterior cingulate gyrus (BAs 24, 32, 33) (*r* = 0.42; *p* = 0.03) (Li et al., 2020). Despite these findings, uncertainty remains regarding the generalizability of these correlations to broader populations, as this study is performed on only *N* = 68 subjects. Further research has also indicated a positive relationship between cortical thickness and volume in the inferior parietal lobe (BAs 39, 40) and FSIQ, as well as performance IQ (PIQ), at a cluster-forming threshold (CFT) of *p* < 0.05 (Bajaj et al., 2018). Similarly, associations between cortical thickness and volume and verbal IQ (VIQ) were found in the left insula (BAs 13, 14, 16) and FSIQ within the inferior frontal gyrus (BAs 44, 45, 47) (Bajaj et al., 2018). However, this study is performed on *N* = 56 subjects, and it is crucial to consider that not all studies may confirm these findings, emphasizing the need for a more nuanced understanding of these relationships. The local gyrification and surface area in the superior parietal (BA 7), left supramarginal (BA 40), left caudal middle frontal (BA 22), left pars opercularis (BA 44), left inferior temporal (BA 20), right inferior and middle temporal (BA 21), right medial orbitofrontal (BAs 11, 12), and right rostral middle frontal (BA 10) regions are also found correlated to *gF* (*r* = 0.29; *p* < 0.001) and (*r* = 0.22; *p* < 0.001), respectively, and to *gC* (*r* = 0.28; *p* < 0.001) and (*r* = 0.28; *p* < 0.001), respectively, on a healthy young dataset (*N* = 740, age = 21-35 years) (Tadayon et al., 2020). However, the reliance on a homogeneous sample of young adults may limit the generalizability of this study to broader age ranges and populations. Additionally, Mullen scales of early learning (MSEL) cognitive ability such as visual reception, fine motor, receptive language, expressive language, and early learning composite, has also been found positively correlated (*r* = 0.14, *p* = 0.025; *r* = 0.186, *p* = 0.002; *r* = 0.147, *p* = 0.016; *r* = 0.120, *p* = 0.049, respectively) with the cortical thickness of the infants at age 1 year, especially in the bilateral superior frontal and middle frontal gyri (BA 10), right medial superior frontal gyrus (BA 10), right occipital superior gyrus (BA 19), bilateral superior parietal cortices (BA 7), left primary motor cortex (BA 4), bilateral anterior cingulate (BAs 24, 32, 33) and precuneus (BA 4), and right superior and middle temporal cortices (BA 22) areas (Girault et al., 2020). Despite these findings, the modest effect sizes (correlation coefficients) suggest that other factors might also play a significant role in early cognitive development, which may limit the explanatory power of cortical thickness alone. Better FSIQ level has also been reported for thinner parietal association cortices, especially left/right inferior parietal (BAs 39, 40) and left/right superior parietal (BA 7) cortices (Squeglia et al., 2013). However, the inverse relationship between cortical thickness and intelligence, as found here, contrasts with other studies linking greater cortical thickness to higher cognitive abilities, raising questions about the consistency of these findings across different cohorts. In other studies, overall FSIQ has been found (Yang et al., 2013; Choi et al., 2008) to correlate (*r* = 0.3~0.7; *p* < 0.01) with the cortical thickness, surface area, sulcal depth, curvature from the left and right parahippocampal gyrus (BA 34), left olfactory cortex (BA 35), right fusiform gyrus (BA 37), bilateral transverse temporal gyri (BAs 41, 42), bilateral thalamus, left parahippocampal gyrus (BA 34), left hippocampus, right opercular part of inferior frontal gyrus (BAs 44, 45, 47), left anterior cingulate gyrus (BAs 24, 32, 33), right amygdala, left lingual gyrus (BA 19), left superior parietal lobule (BA 7), right inferior parietal lobule (BAs 39, 40), left angular gyrus (BA 39), left paracentral lobule, and left caudate nucleus (BAs 1-4). Yet, the wide range of brain areas implicated here raises doubts about the specificity of these findings, as it remains unclear which regions are most critical to the observed associations with intelligence.

#### 5.2.2 Individual-level AI-based predictions

Cortical metrics have also been linked to individual cognitive abilities in AI-based prediction studies. For instance, research using the ABCD dataset predicted residual fluid intelligence (*gF*) scores in over 4,500 adolescents. These studies reported a mean squared error (MSE) between 93 and 95, despite the true residual fluid intelligence scores ranging from−40 to 30 (Oxtoby et al., 2019; Rebsamen et al., 2019; Valverde et al., 2019; Pölsterl et al., 2019a,b; Guerdan et al., 2019). This suggests that although the models demonstrate some predictive power, the magnitude of the error is large relative to the score range, highlighting the need for improved model accuracy. These studies incorporated cortical thicknesses, curvatures, and surface areas alongside regional volumes from various brain structures, including the left middle temporal gyrus (BA 21) and the right superior temporal gyrus (BA 22). However, it is essential to note that while these studies observed significant positive correlations with neurocognitive outcomes, replication studies are needed to validate these findings. Additionally, along with finding a correlation of MSEL cognitive ability with the cortical thickness (discussed in the previous section) of the infants at age 1 year, Girault et al. (Girault et al., 2020) used a linear mixed effect model to predict 2-year neurocognitive scores using cortical metrics such as cortical thickness and cortical surface area. Similar findings are also reported for MSEL-based future (at 4 years of age) cognitive score prediction using sMRI brain features at birth, such as cortical thickness, mean curvature, local gyrification index, vertex area, vertex volume, sulcal depth in string distance and sulcal depth in Euclidean distance with a mean root square error of 0.023–0.18 (Adeli et al., 2019; Zhang et al., 2018, 2020; Cheng et al., 2022, 2023). Furthermore, Wang et al. (2015) used multi-kernel SVR for estimating IQ values using cortical thickness, surface area, sulcal depth, and curvature from BAs 1, 2, 3, 4, 7, 32, 34, 39, 40, 41, 42, 44, 45, and 47 and obtain an average correlation coefficient of 0.718 and a mean root mean square error of 8.695 between the true FSIQs and the estimated ones. Nonetheless, the variability in results underscores the need for careful interpretation of AI-based predictions in cognitive assessment, as further replication and exploration of these relationships are warranted.

#### 5.2.3 Salient brain regions across various neurocognitive measures

**Figure 5:**
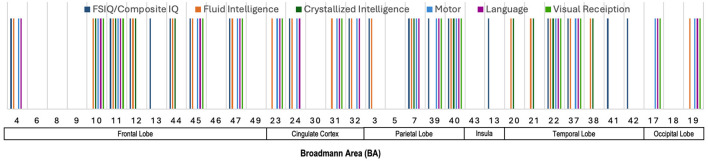
Illustration showing the Broadmann Areas, which have been found salient at least once in one or more reviewed articles, reporting an association of cortical surface metrics with various neurocognitive measures.

Figure 5 presents an illustration of the Broadmann Areas that were identified as salient in studies investigating associations between cortical surface metrics and various cognitive and motor functions. This figure integrates the key findings across multiple studies, offering a visual summary of the brain regions most frequently implicated in significant correlations with a variety of neurocognitive domains. Several regions in the frontal lobe (BAs 4, 10, 11, 12, 45, and 47) show broad involvement in multiple domains, including FSIQ/Composite IQ, fluid intelligence, crystallized intelligence, motor function, and language. Particularly, BAs 4, 10, 11, 45, and 47 are prominently linked to motor functions. The cingulate cortex, including BAs 23, 24, 31, and 32, appears to be involved across a range of domains, including fluid intelligence, motor, and language, suggesting a more generalized role in higher-order cognition. Moving to the parietal lobe (BAs 7 and 40), there is a notable association with FSIQ, fluid and crystallized intelligence, motor, and visual reception. These findings are consistent with the parietal lobe's role in integrating sensory information and spatial reasoning, which underlies various cognitive functions. The temporal lobe regions (BAs 20, 21, 22, 37, and 38) demonstrate associations mostly with fluid intelligence, reinforcing their role in auditory processing, language comprehension, and higher cognitive functions. Lastly, in the occipital lobe (BAs 17 and 19), the involvement of these areas with visual reception, motor, and language is evident, confirming their primary function in visual processing and their role in intelligence through visual-spatial reasoning.

### 5.3 Voxel- and surface-based morphometry to infer neurocognition

Voxel-based morphometry (VBM) (Wright et al., 1995) and surface-based morphometry (SBM) (Kim et al., 2017) allow the correlation of MRI volume or surface metrics at the voxel or surface vertex level (see Supplementary Figure 1). They are extensions of the correlation from the regional or surface area levels into the voxel- or vertex-levels (Whitwell, 2009). In Supplementary Table 3, we summarized existing VBM and SBM-based neurocognitive predictive studies.

#### 5.3.1 Population-level correlation analysis

VBM and SBM methods have provided valuable insights into the neuroanatomical correlates of cognitive abilities. For instance, VBM-based gray matter volumes in the left gyrus rectus (BA 11) and anterior cingulate gyrus (BAs 24, 32, 33), left posterior insula (BAs 13, 14, 16), left superior and middle frontal gyri (BA 10) are found to be positively correlated (*t* score = 4.94; *p* < 0.005) to VIQ scores (Hidese et al., 2020). However, these findings come from studies with relatively small sample sizes (*N* = 266), and further research is necessary to verify the consistency of these results across larger, more diverse cohorts. Similarly, SBM-base shape features in the left inferior and middle temporal (BAs 20, 21), left inferior parietal (BAs 39, 40), and left medial frontal (BA 25) regions showed positive associations (β > 100; *p* < 0.001) with FSIQ (McDermott et al., 2019). Another study (Ramsden et al., 2011) divided their study population into average (FSIQ = [80, 119]), low (FSIQ < 80), and high (FSIQ > 119) groups, and observed that the correlation between the change in VIQ and the change in the gray matter density in the motor area (BAs 4, 6) and anterior cerebellum is 0.876 (*p* < 0.01) for high ability, 0.797 (*p* < 0.05) for average ability and 0.660 (*p* < 0.05) for low ability groups, respectively. Similarly, the corresponding effects were seen for PIQ as 0.492 (*p* > 0.05) for high ability, 0.788 (*p* < 0.05) for average ability, and 0.715 (*p* < 0.01) for low ability groups. These findings highlight that cognitive performance may be differentially associated with neuroanatomical changes depending on baseline cognitive abilities.

#### 5.3.2 Salient brain regions across various neurocognitive measures

**Figure 6:**
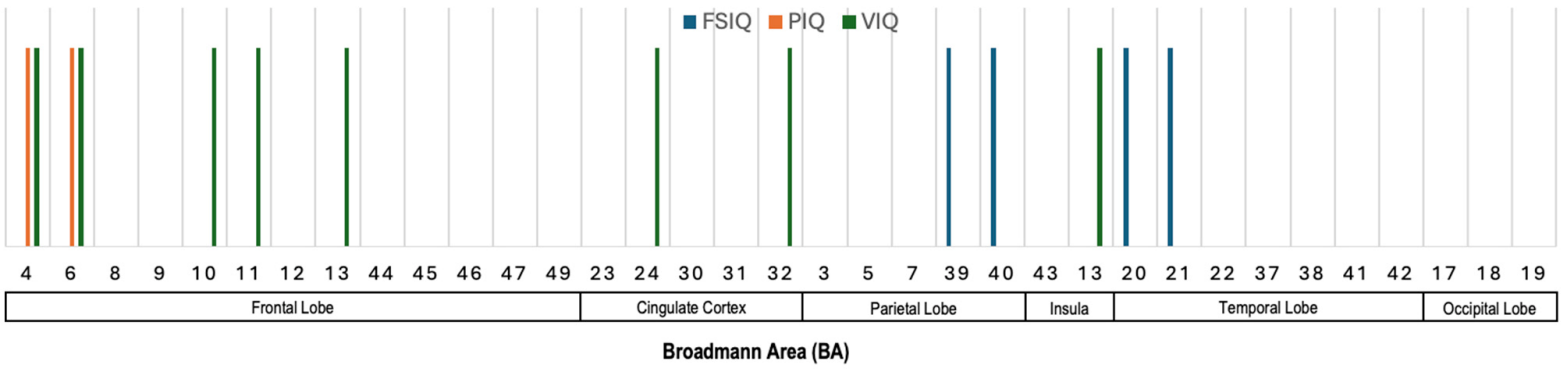
Illustration showing the Broadmann Areas, which have been found salient at least once in one or more reviewed articles, reporting an association of voxel- and surface-based morphometric features with various neurocognitive measures.

Figure 6 illustrates the Broadmann Areas identified as significant in studies examining the associations between voxel- and surface-based morphometric measures and different intelligence scores, specifically FSIQ (or *g*), PIQ (or *gF*), and VIQ (or *gC*). In the frontal lobe, BAs 4 and 6 were consistently linked to FSIQ and PIQ, underlining the frontal lobe's role in both general and performance-related cognitive abilities. Additionally, BA 10, 11, and 13 were associated with VIQ, which supports the frontal lobe's established contribution to verbal reasoning and executive functions. The cingulate cortex, particularly BAs 24 and 32, was predominantly associated with VIQ, reflecting its involvement in attention, emotional regulation, and cognitive processes related to verbal abilities. In the parietal lobe, BAs 39 and 40 were linked to FSIQ, which aligns with the parietal lobe's role in integrating sensory information and supporting higher-order reasoning. These regions are crucial for spatial and mathematical reasoning, processes that are integral to broader measures of intelligence. The insula (BA 13) and the temporal lobe (BAs 20 and 21) were also identified as significant for VIQ and FSIQ. These findings reinforce the temporal lobe's role in auditory processing and language comprehension, both of which are critical for verbal intelligence.

### 5.4 Summary of sMRI and neurocognition

Figures 4–6 illustrate the key Broadmann Areas identified in studies investigating associations between brain volume, cortical surface metrics, and morphometric measures with various neurocognitive functions and intelligence scores. Figure 4 highlights BAs in the frontal lobe (e.g., BAs 4, 6, 8, 9), consistently linked to fluid intelligence and FSIQ, emphasizing the role of the frontal lobe in supporting cognitive functions. BAs in the cingulate cortex (e.g., BAs 23, 31) and the parietal and occipital lobes also correlate with general cognitive abilities. Figure 5 shows multiple BAs in the frontal (e.g., BAs 4, 10, 11), cingulate (BAs 23, 24), parietal (BAs 7, 40), and temporal lobes (e.g., BAs 20, 21) associated with various domains like IQ, motor functions, fluid intelligence, and language, highlighting their roles in motor control, sensory integration, and cognitive processing. Figure 6 focuses on morphometric measures and their links to intelligence scores like FSIQ, PIQ, and VIQ, with the frontal lobe (BAs 4, 6), cingulate cortex, parietal (BAs 39, 40), and temporal lobes associated with reasoning, language, and performance-related intelligence.

## 6 Diffusion MRI to infer neurocognition

Diffusion MRI (dMRI) is a specialized imaging technique that measures the diffusion of water molecules in biological tissues. It provides information about the microstructural organization and integrity of tissues, particularly white matter in the brain. Diffusion tensor imaging (DTI) is a specific type of diffusion MRI technique that assumes a tensor solution to quantify the diffusion properties of water molecules within tissues. It is widely used to investigate the structural connectivity and organization of white matter tracts in the brain. By analyzing the diffusion tensor, various measures can be derived, including fractional anisotropy (FA, which measures the directionality of water diffusion, ranging between 0 for completely isotropic diffusion in all directions and 1 for single-directional diffusion), mean diffusivity (MD, which measures the magnitude of water diffusion), axial diffusivity (AD), which measures the rate of diffusion of water molecules along the principal axis of diffusion, and radial diffusivity (RD), which measures the rate of diffusion of water molecules perpendicular to the principal axis of diffusion.

In most DTI-neurocognition studies in the literature, these maps are used together with a few exceptions with FA to establish the relationship between the integrity of white matter tracks and neurocognition. DTI can also be used to construct tracts, which characterize the major direction of white matter tracts that water flows alongside and is also known as structural connectivity. In Supplementary Table 4, we summarized existing diffusion MRI-based neurocognitive predictive studies.

### 6.1 Population-level correlation analysis

Several studies have demonstrated correlations between FA and neurocognitive function at the population level, though it is important to recognize that replication issues and methodological differences between studies may raise uncertainty about their generalizability. For example, FA has been found to account for 10% of the variance in general intelligence (*g*) (Penke et al., 2012). Specific regions, such as the right anterior thalamic radiation, left superior longitudinal fasciculus, left inferior frontal-occipital fasciculus, and left uncinate fasciculus (BAs 1, 3-9, 11, 13, 17, 18, 22, 24, 25, 29, 32, 34-36, 38, 39, 41, 42-47), have shown significant correlations with FSIQ (*r* = 0.53; 95% CI 0.35–0.66) (Malpas et al., 2016). Additionally, FA in the corpus callosum (*r* = 0.48; *p* < 0.003) (Navas-Sánchez et al., 2014), the medial orbital frontal cortex (BA 25) (*r* = 0.496, *p* = 0.01 (Nestor et al., 2015); *r* = 0.463, *p* = 0.020 (Ohtani et al., 2017)), and the right inferior frontal-occipital fasciculus (*p* = 0.05) (Wang et al., 2012) have all been found to correlate with FSIQ. However, it is crucial to acknowledge that while these findings are promising, they represent only specific studies. The question remains whether they can be reliably replicated, as other studies may report different effect sizes or even null results depending on sample characteristics and methods. Similarly, studies like those conducted by Clayden et al. (2012) have shown that the third principal component of FA, estimated across different tracts, is predictive of FSIQ (*F* = 8.36, *P* < 0.01) and that the second principal component of MD predicts FSIQ (*F* = 4.60, *P* < 0.05). However, the applicability of these models to different populations has not been rigorously tested, raising concerns about generalizability. Further population-level studies have also shown that *gF* has the strongest correlation with FA (*r* = 0.57) (Haász et al., 2013) and that whole-brain mean FA is positively correlated with emotional processing (*r* = 0.63; *p* < 0.05) (Pisner et al., 2017). In one population-based study, FA and AD were greater in individuals with higher IQs (FSIQ > 130) than in a control group, especially in widespread white matter regions associated with frontal, central, and associative pathways (Nusbaum et al., 2017). These population-level findings suggest widespread relationships between FA and various cognitive measures, but replication efforts remain important to confirm the consistency of these effects. In developmental studies, FA at 2 weeks of age correlated with neurodevelopmental outcomes at 2 years of age (*r* = 0.35–0.48) (Feng et al., 2019). Pearson correlation analysis also showed a negative relationship between VIQ (*gC*) and FA in the left-hemispheric Broca's area (*r* = −0.73; *p* < 0.001) (Konrad et al., 2012), while MD in the same region correlated positively with VIQ (Konrad et al., 2012). Although these findings highlight the potential of FA as a marker for early brain development, using such correlations to predict future outcomes is still in its infancy. Longitudinal studies with larger sample sizes and varied populations would be necessary to determine these early correlation's robustness. Finally, FA has also been used in studies exploring neurodegenerative conditions, such as a study that found significant relationships between FA and chronic neurological damage in retired National Football League players. This study showed that ~24% of participants demonstrated neurophysiological impairments based on Mini-Mental State Examination (MMSE) evaluation (Casson et al., 2014). Again, although this study is an important early step, further population-level investigations are necessary to assess the consistency of these findings across different samples. Several other population-level investigations have explored the relationship between white matter diffusion metrics such as FA, AD, and RD, and various cognitive outcomes. For example, Lee et al. (2017) conducted a study as part of the UNC-Chapel Hill Early Brain Development Study and examined the correlation between AD, RD, and FA with early learning composite (ELC) scores in a population of infants aged 0–2 years. They reported correlation coefficients ranging from 0.13 to 0.20 (*p* < 0.05) for these diffusion metrics, suggesting a modest but significant relationship between white matter development and early cognitive function. Similarly, Dunst et al. studied a sample of 63 adults aged 18–50 years in Austria and used FA and RD metrics to investigate their relationship with intelligence scores obtained from the Intelligence Structure Battery (INSBAT) (Dunst et al., 2014). However, no significant group differences in FSIQ were observed between the sexes, highlighting the potential variability of FA-intelligence relationships across demographic subgroups. In older populations, Fischer et al. examined the correlation between FA and FSIQ in a cohort of 43 elderly individuals aged 60–85 years (Fischer et al., 2014). Their results indicated that while younger elderly participants showed slightly higher FSIQ than their more advanced-aged counterparts, the difference was not statistically significant, suggesting that the relationship between white matter integrity and cognitive function may attenuate with age. Among pediatric populations, Nusbaum et al. compared children with higher IQs (FSIQ > 130) to a control group in a study of 44 participants aged 8–12 years in France (Nusbaum et al., 2017). Their results showed greater AD and FA values in widespread white matter regions associated with frontal, central, and associative pathways in the higher IQ group, providing further evidence of the relationship between white matter development and intelligence. Koenis et al. (2018) explored brain network efficiency in a large sample of 330 individuals aged 9-23 years from the Netherlands Twin Register. They found that FSIQ at age 18 was positively correlated (*r* = 0.28; *p* < 0.0001) with global brain network efficiency as measured by FA-weighted brain networks, suggesting that network efficiency may play a role in cognitive functioning during late adolescence. Additionally, Ponsoda et al. (2017) used tractography-based brain connectivity matrices in a study of 94 young adults (mean age 20.0 ± 1.7 years) from Spain. They found that individuals with similar brain connectivity profiles were also more similar in their levels of *gF* and *gC*, further supporting the notion that white matter connectivity is linked to cognitive abilities. Kenett et al. (2018) examined the relationship between anatomical connectivity and cognitive performance using tractography and parcellation in a sample of 416 young adults from the United States. Their study focused on the inferior parietal lobe and found a positive correlation (*r* = 0.11; *p* < 0.02) between average controllability and cognitive performance as measured by the Combined Raven's Test (CRT), suggesting that regional white matter properties may contribute to specific cognitive abilities. Lastly, Kocevar et al. (2019) conducted a study using tractography-based brain connectivity matrices in 43 children aged 8–12 years and found that global brain connectivity was strongly associated with high intelligence scores. These findings add further support to the idea that brain network homogeneity may be a marker of cognitive abilities in both children and adults.

### 6.2 Individual-level AI-based predictions

While population-level studies offer insight into broad trends, AI-based approaches have gained attention for their ability to make individualized predictions about neurocognitive function. AI models have been used to predict cognitive outcomes based on structural and diffusion MRI features, including FA, AD, and MD. For instance, one study employed DTI features from the whole brain, such as connected surface area (CSA), weighted CSA, FA, MD, and cluster number in a latent partial multi-view multitask representation learning, to predict *gF*, reporting a correlation between actual and estimated *gF* of 24.11% (*p* < 0.001) (Zhang et al., 2019).

### 6.3 Salient brain regions across various neurocognitive measures

**Figure 7:**
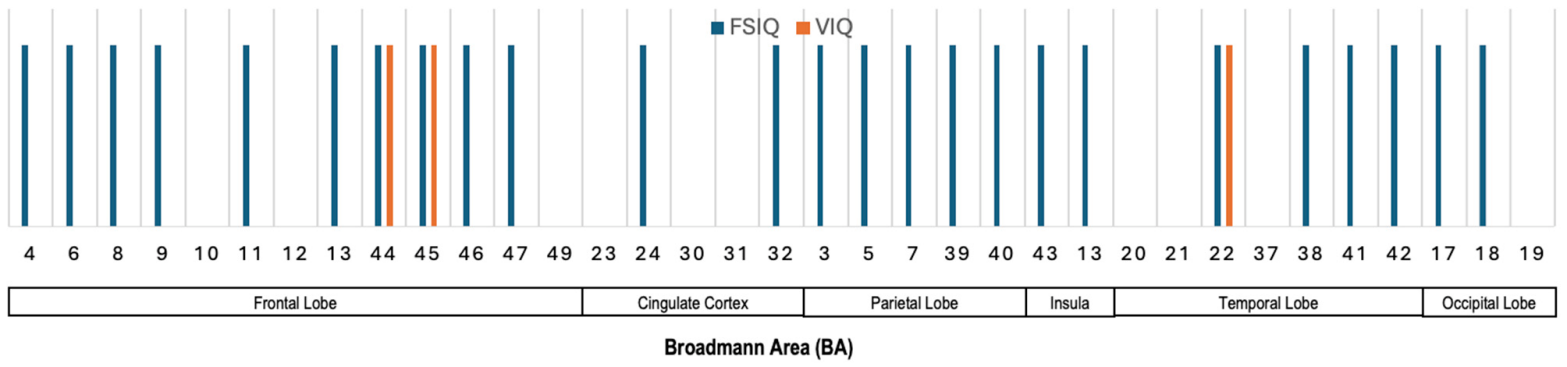
Illustration showing the Broadmann Areas, which have been found salient at least once in one or more reviewed articles, reporting an association of dMRI features with various neurocognitive measures.

Figure 7 presents the Broadmann Areas identified as significant in studies investigating associations between dMRI features and various neurocognition scores. As most of the dMRI-based approaches reviewed in this study do not specify specific brain regions that can be translated to multiple Broadmann areas (see Supplementary Table 4), we use information from studies that specified salient Broadmann areas that connect to different neurocognition functions. We see in Figure 7 that several BAs (4, 6, 8, 9, 11, 13, 44, 45, 46, and 47) in the frontal lobe are linked predominantly to FSIQ, with BA 44 and BA 45 also showing an association with VIQ. BAs 24 and 32 in the cingulate cortex are associated with FSIQ. In the parietal lobe, BAs 3, 5, 7, and 39 show connections with FSIQ. Notably, BA 22 in the temporal lobe demonstrates associations with both FSIQ and VIQ, highlighting their roles in cognitive and verbal processing. Finally, BAs 17 and 18 in the occipital lobe are linked to FSIQ, emphasizing the occipital lobe's involvement in visual processing and spatial reasoning.

### 6.4 Summary of dMRI and neurocognition

The population-level correlation analysis reveals that fractional anisotropy (FA) in specific brain regions is consistently linked to neurocognitive function, particularly general intelligence (*g*). Key areas include the right anterior thalamic radiation, superior longitudinal fasciculus, inferior frontal-occipital fasciculus, and uncinate fasciculus, as well as the corpus callosum and medial orbital frontal cortex. These regions are associated with various cognitive measures, though replication challenges raise questions about their generalizability. Studies show significant correlations between FA and intelligence, emotional processing, and neurodevelopment, with specific brain networks and white matter regions (e.g., frontal, parietal, temporal, and occipital lobes) linked to cognitive abilities like FSIQ, PIQ, and VIQ. While findings are promising, more research is needed to confirm the consistency of these relationships across different populations. Figure 7 further highlights the involvement of frontal (e.g., BAs 4, 6, 8, 9, 44, and 45), cingulate (BAs 24, 32), parietal (BAs 3, 5, 7, and 39), temporal (BA 22), and occipital lobes (BAs 17, 18) in supporting various cognitive functions, reinforcing their importance in neurocognitive processing.

## 7 Functional MRI to infer neurocognition

Functional MRI (fMRI) utilizes the blood oxygenation level-dependent (BOLD) effect to reveal brain connectivity during a resting state (rs-fMRI) (Gore et al., 2019) (see Supplementary Figure 3). Major large-scale brain networks as found in resting-state fMRI include the dorsal and ventral default mode, right and left executive control, dorsal and ventral attention, anterior and posterior salience, basal ganglia, language, high and primary visual, precuneus, auditory and somatosensory networks, and others (Shirer et al., 2012). Functional connectivity strength among different parts of the brain as estimated from fMRI was found to be associated with neurocognitive and intelligence levels in humans (Vakhtin et al., 2014; Schultz and Cole, 2016; Kruschwitz et al., 2018; Pezoulas et al., 2017; Noble et al., 2017).

### 7.1 Population-level correlation analysis

As summarized in Supplementary Table 5, Vakhtin et al. (2014) conducted a study that showed that functional brain networks, covering attentional, cognitive, default-mode, sensorimotor, visual, auditory, and basal ganglia regions remained stable across resting-state and complex cognitive tasks. These findings indicate a consistency in network spatial features when transitioning between different brain states. In another study, Schultz and Cole (2016) observed that high-performing individuals demonstrated more efficient brain connectivity updates, as reflected in smaller changes to the functional network architecture between rest and task states, with Pearson correlation measures used to assess network similarity. Kruschwitz et al. (2018) analyzed 1,096 participants but found no significant association between characteristic path length and global efficiency. This suggests that these metrics may not be robust indicators of network functionality in this context. Pezoulas et al. (2017) studied the cerebellum's functional connectivity in 136 participants. They identified sex-based differences in IQ: high-IQ females had significantly higher average clustering coefficients and characteristic path length than high-IQ males, highlighting potential gender-specific variations in brain connectivity patterns. In another study, Noble et al. (2017) demonstrated a 22% correlation between actual and estimated fluid intelligence (*gF*) (*p* < 0.0001) based on connectivity patterns in 10 functionally coherent networks across the whole gray matter, as assessed through Raven's Progressive Matrices. Song et al. (2008) performed a population-level correlation analysis, showing that functional connectivity in the bilateral dorsolateral prefrontal cortices (BA 9) was significantly correlated (*r* = 0.47; *p* = 0.0002) with Wechsler Adult Intelligence Scale (WAIS) scores. Similarly, frontoparietal regions have been implicated in various neurocognitive measures, such as FSIQ (*g*), general fluid intelligence (*gF*), and crystallized intelligence (*gC*). Regional homogeneity of functional connectivity in frontoparietal and central brain regions (BAs 1, 2, 3) has been shown to correlate with FSIQ (Wang et al., 2011; Langeslag et al., 2013; Basten et al., 2013; Pamplona et al., 2015; Hilger et al., 2017a,b), *gF* (Hearne et al., 2016; Santarnecchi et al., 2017), and *gC* (Hearne et al., 2016) performance. These correlations, while promising, often vary in effect size, with some studies reporting lower correlations that may reflect sample-specific characteristics. Notably, the strength of these associations suggests that frontoparietal connectivity may serve as a biomarker for cognitive abilities, although replication efforts remain necessary to confirm these effects across diverse populations. Frontoparietal network integrity, particularly in BAs 4, 7, 11, 12, 13, 14, 16, 24, 32, 33, and 40, has also been linked to fluid intelligence (*gF*) (Ebisch et al., 2012). Connectivity in the lateral prefrontal cortex (BAs 9, 10, 46) has been correlated with *gF* (*r* = 0.28–0.32; *p* = 0.006–0.0015) (Cole et al., 2012; Cole M. W. et al., 2015). As a result, many diseases [e.g., Turner syndrome (Hart et al., 2006)] related to impairment in the frontoparietal network are also associated with a deficit in the *gF*/VIQ, compared to a healthy population (*p* < 0.0001). Some other studies used fMRI-based functional connectivity data from the Human Connectome Project (HCP) to show a correlation between the actual and estimated fluid intelligence (*r* = 0.19–33) (Greene et al., 2018; Elliott et al., 2019; He et al., 2018; Li et al., 2018; Dubois et al., 2018a) and cognitive ability (*r* = 0.95) (Yoo et al., 2019). Further, when compared between average IQ and higher IQ healthy population, greater BOLD activation across different brain regions, including parietal, caudate, fusiform, and occipital areas (BAs 3, 4, 6, 7, 8, 9, 19, 31, 32, 38, 46, 47), is seen for complex reasoning in higher IQ population (Graham et al., 2010).

### 7.2 Individual-level AI-based predictions

Additionally, functional connectivity in the frontoparietal network has been used for predicting later-life neuropsychological performance, with correlations (*r*) ranging from 0.08 to 0.44 (*p* < 0.001) (Kwak et al., 2021). Despite these correlations, the predictive accuracy of functional connectivity measures for behavioral outcomes may vary across studies and populations, introducing uncertainty about the generalizability of these findings. Other studies have focused on specific networks, such as the frontoparietal network (BAs 9, 4, 39, 40, 46, 10, 13, etc.), which has been significantly correlated with fluid intelligence (*r* = 0.50; *p* < 0.01) (Finn et al., 2015), memory (*r* = 0.097; *p* < 0.001) (Powell et al., 2018), general neurocognitive ability (*r* = 0.31; *p* < 0.0001) (Sripada et al., 2020), and FSIQ (*r* = 0.51; *p* < 0.001) (Jiang et al., 2017) performance.

### 7.3 Salient brain regions across various neurocognitive measures

**Figure 8:**
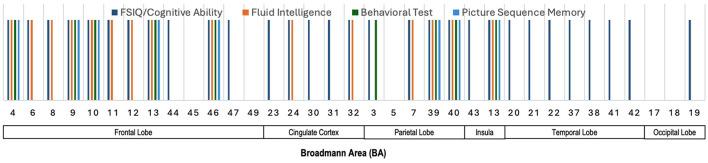
Illustration showing the Broadmann Areas, which have been found salient at least once in one or more reviewed articles, reporting an association of fMRI features with various neurocognitive measures.

Figure 8 illustrates the Broadmann Areas identified in studies investigating associations between fMRI features and cognitive functions, including FSIQ/Cognitive Ability, fluid intelligence, behavioral test performance, and Picture Sequence Memory. Many regions in the frontal lobe, such as BAs 4, 6, 8, 9, 10, 11, 12, 13, 44, and 46, are associated with a wide range of functions. FSIQ/Cognitive Ability and fluid intelligence (BAs 4, 6, 8, 9, 10, 11, 12, 13, and 46) dominate, while Picture Sequence Memory and Behavioral tests also show a role in BAs 4, 9, 10, 13, and 46. BAs 23, 24, 30, 31, and 32 in the cingulate cortex exhibit involvement across fluid intelligence, behavioral testing, and FSIQ/Cognitive Ability. In the parietal lobe, BAs 3, 7, 39, and 40 are notably associated with multiple cognitive domains, including FSIQ/Cognitive Ability, fluid intelligence, behavioral test performance, and Picture Sequence Memory. These areas are crucial for processing sensory input and spatial reasoning, consistent with their broad involvement. BA 13 in the Insula is notably associated with FSIQ/Cognitive Ability, fluid intelligence, behavioral test performance, and Picture Sequence Memory. Temporal lobe BAs 20, 21, 22, 37, 38, 41, and 42 are primarily linked to FSIQ/Cognitive Ability, reinforcing their role in memory and higher cognitive functions. BA 19 in the occipital lobe shows involvement primarily with FSIQ/Cognitive Ability, confirming their key role in visual processing and spatial reasoning.

### 7.4 Summary of fMRI and neurocognition

The population-level correlation analysis underscores the importance of consistent functional brain network activity across resting-state and cognitive tasks, with key regions including the frontoparietal, cingulate, and temporal lobes. Several studies demonstrate that efficient brain connectivity, particularly in the frontoparietal network (BAs 9, 10, 46), is linked to cognitive measures such as FSIQ, fluid intelligence (*gF*), and crystallized intelligence (*gC*). The correlations between functional connectivity and cognitive abilities, though varying in effect sizes, suggest that frontoparietal connectivity could serve as a biomarker for neurocognitive performance. These findings are consistent across multiple brain regions, including the dorsolateral prefrontal cortex, cingulate cortex, and insula, all showing significant associations with cognitive functions. Figure 8 highlights the key brain regions involved in cognitive abilities, with the frontal, parietal, and temporal lobes playing central roles in FSIQ, fluid intelligence, and behavioral performance, reinforcing their importance in neurocognitive processing. While these correlations are promising, further replication studies are necessary to confirm the robustness of these associations across diverse populations.

## 8 Deep features from MRI to infer neurocognition

**Figure 9:**
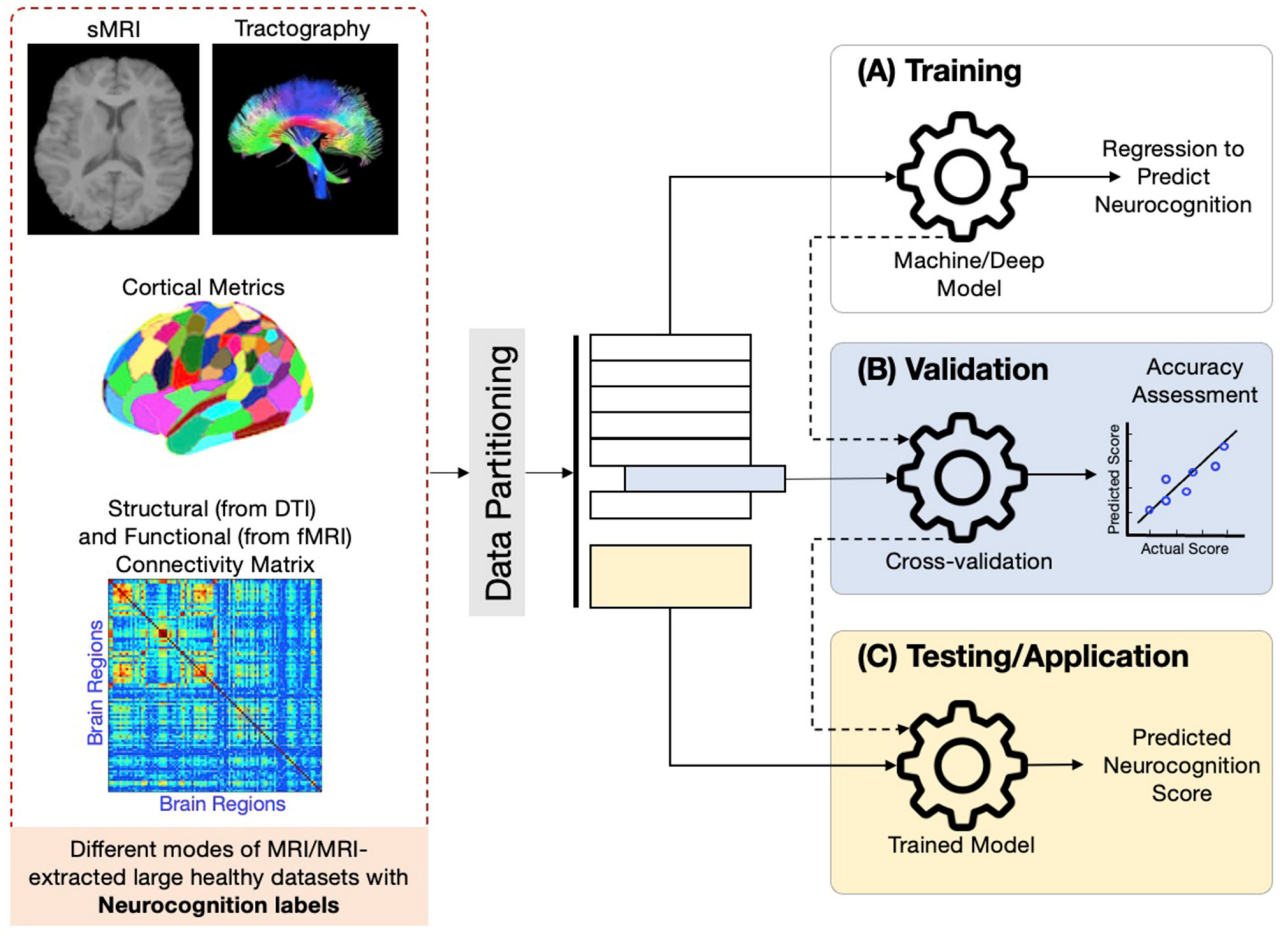
Typical machine/deep learning inferring neurocognition working pipeline that uses large brain datasets from various MRI modalities to predict neurocognitive/intelligence scores.

Regional, surface-area, voxel, and vertex-level features are so-called handcrafted or hand-engineered features. They carry neuroanatomic meanings and are easy to interpret. On the other hand, deep learning extracts tens of thousands or even millions of “deep features” from the whole MRI or image patches. Those deep features are extracted from convolutions of images with filters (3 × 3 × 3, 5 × 5 × 5, or other sizes), or the so-called attention mechanisms in vision transformer (ViT) deep learning models. In Figure 9, we show a working pipeline of machine/deep learning approaches that make use of different modes of brain MRI or MRI-extracted hand-engineered data to predict neurocognition/intelligence scores for each subject.

### 8.1 Individual-level AI-based predictions

Several studies (Chiang et al., 2019; Ranjbar et al., 2019; Vang et al., 2019; Pominova et al., 2019; Zou et al., 2019; Liu et al., 2019) used convolutional neural networks (CNNs), a specific type of image-based deep learning technique, on T1-MRI to predict fluid intelligence (*gF*) in adolescents. They predicted the residual fluid intelligence score of more than 4,500 adolescents with a mean square error (MSE) ranging from 92 to 103, the true residual fluid intelligence scores ranged from −40 to 30, as summarized in Supplementary Table 6. However, the interpretation of deep features is difficult. A potential solution is to choose brain regions beforehand and input those regions into deep learning models. For example, Zou et al. (2019) used regions from bilateral transverse temporal gyri (BAs 41, 42), bilateral thalamus, left parahippocampal gyrus (BA 34), left hippocampus, right opercular part of inferior frontal gyrus (BAs 44, 45, 47), left anterior cingulate gyrus (BAs 24, 32, 33), right amygdala, left lingual gyrus (BA 19), left superior parietal lobule (BA 7), right inferior parietal lobule (BAs 39, 40), left angular gyrus (BA 39), left paracentral lobule, and left caudate nucleus (BAs 1–4) in their deep learning model to predict *gF* score. However, the choice of such regions may be subjective, the accuracy of prediction was not significantly different from inputting the whole image, and treating regions separately may miss the opportunity to consider those regions jointly in the convolutions. Interpretation of deep learning models can be also achieved by masking or replacing different regions, adding random noise to images, or calculating the saliency, activating, or attention maps (Arrieta et al., 2020; Gunning et al., 2019; Speith, 2022). Their use in interpreting deep learning prediction of intelligence or neurocognition is yet to be studied.

### 8.2 Salient brain regions across various neurocognitive measures

**Figure 10:**
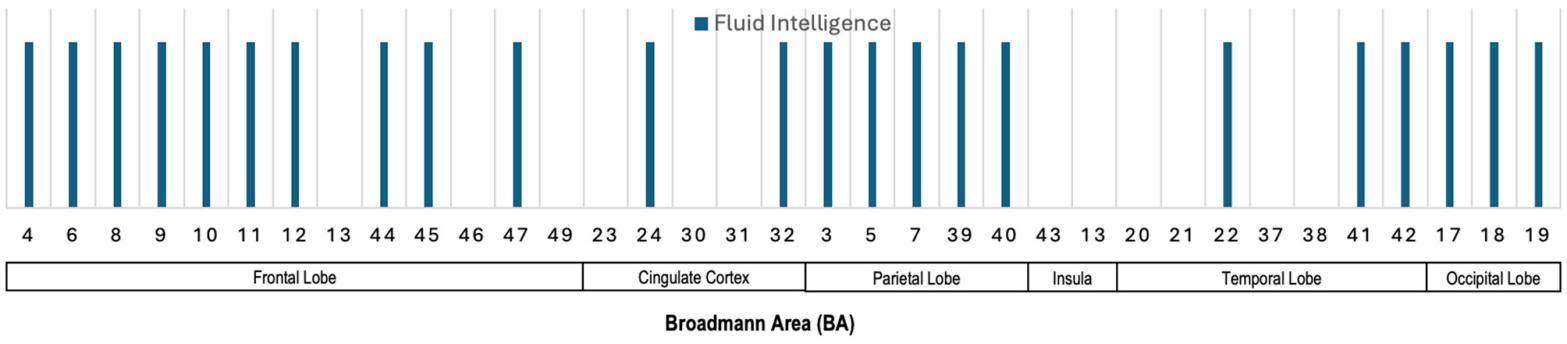
Illustration showing the Broadmann Areas, which have been found salient at least once in one or more reviewed articles, reporting an association of MRI features used in deep learning approaches with various neurocognitive measures.

Despite the challenge of the interpretability of the deep models, Figure 10 presents the specific Broadmann Areas identified as significantly correlated with fluid intelligence when deep models are utilized for the predictive tasks. In the frontal lobe, BAs 4, 6, 8, 9, 10, 11, 12, 44, 45, and 47 show notable involvement in fluid intelligence, supporting the role of executive function, reasoning, and problem-solving typically associated with these areas. The cingulate cortex, including BAs 24 and 32, also demonstrates involvement, which is consistent with the region's role in cognitive control and emotional regulation, both essential for adaptive reasoning and fluid intelligence. In the parietal lobe, BAs 3, 5, 7, 39, and 40 are linked with fluid intelligence, reflecting the importance of sensory integration, attention, and spatial processing in tasks requiring fluid reasoning. The temporal lobe's involvement is indicated by BAs 22, 41, and 42, regions linked to language processing and memory, highlighting the role of these cognitive functions in fluid intelligence. Additionally, regions in the occipital lobe (BAs 17, 18, and 19) show associations with fluid intelligence, underscoring the contribution of visual processing and visual-spatial reasoning to intellectual tasks requiring adaptability and novel problem-solving. This broad distribution of cortical involvement across frontal, cingulate, parietal, temporal, and occipital regions illustrates the multifaceted nature of fluid intelligence, engaging a wide network of brain areas.

### 8.3 Summary of deep learning of MRI and neurocognition

Several studies have utilized CNNs on T1-weighted MRI data to predict fluid intelligence (*gF*) in adolescents, achieving mean square error (MSE) values ranging from 92 to 103 across over 4,500 subjects. However, interpreting deep learning models remains a challenge. One approach to improve interpretability involves pre-selecting specific brain regions for model input, though this method may not significantly improve prediction accuracy and can overlook interactions between brain regions. Techniques like saliency maps and attention mechanisms offer potential solutions for interpreting deep learning models, but their application to intelligence predictions is still under-explored. Despite the challenges in interpreting deep models, certain Broadmann Areas (BAs) have been consistently associated with fluid intelligence across predictive tasks. Frontal areas (BAs 4, 6, 8, 9, 10, 11, 44, 45, 47) are linked to executive function and problem-solving, while cingulate cortex regions (BAs 24, 32) are involved in cognitive control and emotional regulation. Parietal regions (BAs 3, 5, 7, 39, 40) contribute to sensory integration and spatial reasoning, and temporal (BAs 22, 41, 42) and occipital areas (BAs 17, 18, 19) are crucial for language, memory, and visual-spatial processing. This broad cortical distribution underscores the complex, multi-regional brain activity underlying fluid intelligence.

## 9 Reviewed paper's overall agreement with the P-FIT model

**Figure 11:**
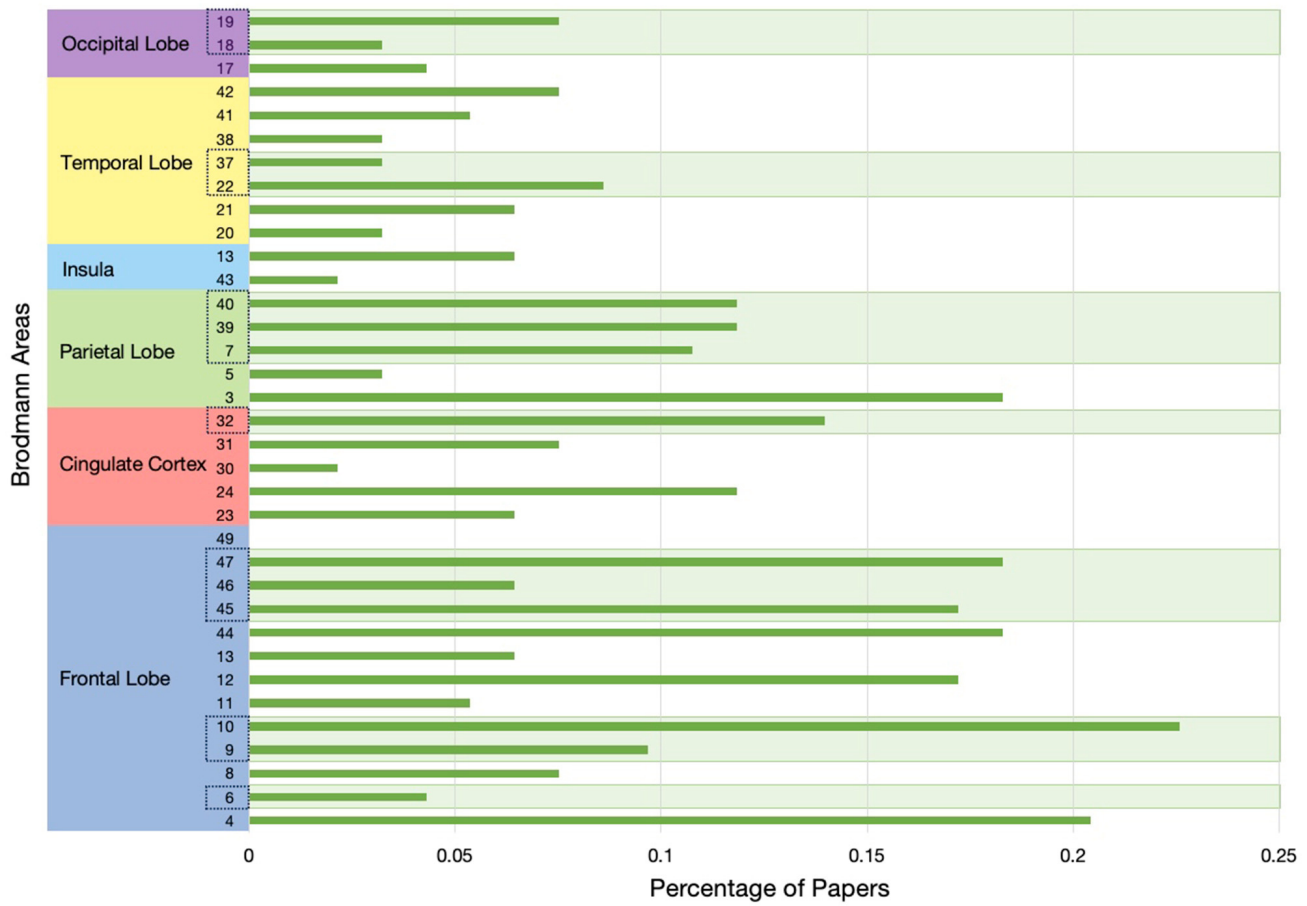
Bar plot representing the percentage of our reviewed papers in this study that used different Brodmann areas (BAs) in inferring intelligence and neurocognition. BA numbers in dotted boxes are part of the P-FIT model. Brodmann areas are not specified for either the left or right hemisphere. Studies that did not specify BAs are also included in the normalization/denominators.

Figure 11 shows a bar plot representing the percentage of our reviewed papers (*N* = 94) that used different Brodmann areas in inferring intelligence and neurocognition. For simplicity, we only used the Brodmann areas without mentioning the hemisphere. We see in this figure that most of the reviewed studies emphasized the frontal lobe, cingulate cortex, and parietal lobe as being influential on human neurocognition and intelligence. In addition, we also see in this figure that most of the reviewed papers found a strong relation between frontal and parietal lobes with the human intelligence and these two regions are a major part of the P-FIT model.

## 10 Opportunities, challenges, and possible solutions

### 10.1 Precision and individual variability

Most of the earlier studies associated MRI metrics with neurocognition in a population, while a smaller number of studies aimed to predict neurocognition at the individual level. The population-level association does not explain individual variability. There is a need to use MRI to estimate or predict neurocognition for individual subjects. A fundamental question remains open for which MRI metrics, out of hundreds of s/d/fMRI metrics, carry the neurocognitive information for individual differences. The answers to this question may vary by the neurocognitive domains. In short, while MRI metrics have been associated with neurocognition at a population level, they fall short in explaining individual variability, and the challenge lies in identifying which specific MRI metrics and neuroanatomical regions carry the neurocognitive information that accounts for individual differences. To address the challenge of identifying which MRI metrics best predict individual differences in neurocognitive outcomes, several approaches can be utilized:

#### 10.1.1 Multimodal fusion approaches

Combining multiple MRI modalities (e.g., s/d/fMRI) into a single predictive framework can help capture a broader range of neurocognitive information. Techniques such as canonical correlation analysis (CCA) (Yang et al., 2019), multiview learning, or deep multimodal networks enable the integration of diverse features from different MRI types, which may enhance the ability to explain individual variability in neurocognitive performance.

#### 10.1.2 Feature selection and explainability

Identifying the most informative MRI features for individual prediction is crucial. Feature selection methods like recursive feature elimination, LASSO, or elastic net regression can help filter out irrelevant or redundant features, focusing on those that carry the most neurocognitive information. Additionally, explainable machine learning methods, such as SHapley Additive exPlanations (SHAP) (Nohara et al., 2019) or local interpretable model-agnostic explanations (LIME) (Mishra et al., 2017), can provide insights into how specific features from different brain regions contribute to individual predictions.

#### 10.1.3 Domain-specific neurocognitive prediction

The relationship between MRI metrics and neurocognition may vary across different cognitive domains (e.g., working memory, attention, fluid intelligence). Using domain-specific models, rather than global models, can help fine-tune the prediction process by focusing on the relevant brain metrics and neuroanatomical regions associated with each specific domain of neurocognitive function.

#### 10.1.4 Longitudinal and personalized modeling

Individual variability in neurocognition may not be fully captured by cross-sectional data. Longitudinal data, where brain changes are tracked over time, can provide a more dynamic understanding of individual neurocognitive trajectories. Personalized modeling approaches, such as reinforcement learning or few-shot learning, could be used to fine-tune predictions for individual subjects based on their unique brain metrics and cognitive profiles.

### 10.2 Neuroscientific interpretation

Sex differences exist widely in diseases (Kanaya et al., 2002) and normal brain MRIs (Cosgrove et al., 2007). Hemispheric differences exist and contribute to brain development. Besides sex and hemispheric differences, brain development presents spatial and temporal heterogeneity. Spatially, maturation occurs in a posterior-to-anterior and inferior-to-superior direction (Sowell et al., 2004; Giedd et al., 1999). Temporally, sensory and motor cortices develop earlier, while the prefrontal, amygdala, and hippocampus mature during adolescence (Giedd et al., 1999; Herting et al., 2018), and working memory (Nagel et al., 2013) and reasoning (Vendetti et al., 2015) evolve over childhood and adolescence. Yet, it remains open to “localizing” the regional brain biomarkers in space, in time, and specific to sex, age, race, ethnicity, and brain hemisphere. Elucidating the neural substrate of inter-individual intelligence difference will also differ across neurocognitive domains. Thus, the challenge remains to localize the regional brain biomarkers specific to sex, age, race, ethnicity, and brain hemisphere, and to elucidate the neural substrate of inter-individual intelligence differences across various neurocognitive domains. Localizing brain biomarkers that account for differences in sex, age, race, ethnicity, and hemisphere, as well as inter-individual intelligence variation across neurocognitive domains, is a complex challenge that can be tackled with the following approaches:

#### 10.2.1 Sex- and age-specific modeling

To account for sex and age differences, sex-stratified models and age-specific developmental models can be employed. These models could analyze MRI metrics separately for males and females, and across age groups, to identify biomarkers specific to sex- and age-related neurodevelopmental trajectories. Machine learning algorithms that incorporate interaction terms can be useful to capture how brain structure-function relationships differ by sex or change over time during key developmental stages, such as childhood, adolescence, and adulthood.

#### 10.2.2 Multivariate and spatiotemporal modeling

The spatial and temporal heterogeneity of brain development requires sophisticated multivariate models that can capture the dynamics of regional brain maturation. Methods such as spatiotemporal graph convolutional networks (ST-GCNs) or longitudinal growth models can integrate spatial and temporal dimensions of MRI data, identifying how different regions mature at different rates and how these changes relate to cognitive functions. These models can also help pinpoint regional brain biomarkers specific to developmental windows and brain hemispheres.

#### 10.2.3 Cultural and ethnic considerations in neuroimaging

Accounting for race and ethnicity in neuroimaging studies requires the inclusion of diverse datasets to avoid the bias often seen for homogeneous samples. Meta-analytic approaches, which combine data from multiple populations, can help identify universal vs. population-specific brain biomarkers. Additionally, transfer learning and domain adaptation techniques can be employed to adapt models trained on one population to another, ensuring that biomarkers are not biased toward a specific ethnic group.

#### 10.2.4 Domain-specific biomarker identification

Since the neural correlates of intelligence are likely to vary across different neurocognitive domains (e.g., working memory, reasoning, verbal intelligence), domain-specific models can help localize which regions are important for specific cognitive functions. This could be achieved using task-specific fMRI paradigms alongside multi-domain modeling frameworks. By differentiating between cognitive domains, researchers can better elucidate the regional brain biomarkers that drive performance in each area of cognition.

#### 10.2.5 Cross-domain integration for personalized neurocognitive profiles

To fully capture the inter-individual differences in intelligence, an integrated approach that combines sex, age, race, hemisphere, and cognitive domain information is essential. Multidimensional models that integrate these factors can reveal more personalized neurocognitive profiles, linking specific brain biomarkers to intelligence across diverse populations. Techniques like individualized prediction models or dynamic functional connectivity analysis could help in developing highly personalized biomarkers for neurocognitive function.

### 10.3 Challenges of interpretability in deep learning methods

Deep learning methods, particularly CNNs and ViTs, have proven powerful in extracting high-dimensional features from MRI data to predict neurocognitive outcomes. However, a major limitation of deep learning models is their lack of interpretability, often referred to as the “black box” problem. While traditional machine learning approaches, such as those based on handcrafted neuroanatomic features, allow for straightforward interpretation grounded in neurobiology, deep learning models extract features that do not necessarily have an obvious neuroanatomic or cognitive meaning. One of the primary challenges is the sheer complexity and scale of the deep features extracted from MRI images. These features are typically low-level pixel representations or highly abstracted patterns derived through multiple layers of convolutions or attention mechanisms, which complicates the task of mapping them back to interpretable brain structures or functions. As a result, the ability to understand *how* deep learning models arrive at their predictions, whether they are predicting intelligence scores or other neurocognitive outcomes, becomes limited. To address this issue, explainable machine learning frameworks have been developed (Pat et al., 2023) to increase both prediction accuracy and interpretability. For example, a recent study applied an explainable machine learning approach to predict cognitive abilities from task-based fMRI during a working memory task in the ABCD cohort (*N* = 3,989). This framework compared multiple predictive algorithms, including Elastic Net, which demonstrated either similar or better prediction performance compared to more complex nonlinear models. Importantly, the study used techniques such as SHAP, Accumulated Local Effects, and Friedman's H-statistic to interpret *how* these algorithms drew information from the brain to make their predictions. These tools helped explain the relative importance of different brain regions in predicting cognitive abilities, providing a clearer picture of the underlying brain-cognition relationships. Another set of interpretability tools includes methods such as saliency maps, activation maps, and attention maps, which highlight the regions of the input image that most strongly influence the model's predictions. These techniques can help researchers identify which brain regions or features are driving the model's decision, providing a degree of interpretability. For example, saliency maps can show which areas of an MRI scan are most critical for predicting intelligence, allowing researchers to verify whether these regions align with known neuroanatomic correlates of cognition. Similarly, occlusion tests, where certain parts of the image are masked or perturbed to assess their contribution to the model's prediction, can offer insights into the model's reasoning. While these tools provide useful insights, their effectiveness remains limited in neuroimaging studies due to the complex and distributed nature of brain functions. Interpretation methods often identify large, diffuse areas of the brain, making it difficult to establish clear links between model features and specific neurocognitive processes. Additionally, these methods lack standardization, and different techniques may yield inconsistent results, raising concerns about their reliability for interpreting deep learning models in cognitive neuroscience. Overall, while interpretability remains a significant challenge in applying deep learning to neuroimaging, the development and application of methods such as region selection, saliency maps, and perturbation-based analyses represent promising directions. However, these techniques are still in their infancy when it comes to predicting neurocognitive outcomes, and further validation is necessary to ensure they provide biologically meaningful insights.

### 10.4 Nature and nurture beyond MRI data

A mystery is to which extent is human intelligence or neurocognition decided by nature (i.e., genetics) and by nurture. For nurture, social upbringing (Steffener et al., 2016) and environment (Hackman et al., 2021) both have effects on neurodevelopment, so do demographics [age, sex, body mass index (BMI), etc.], lifestyle (smoking, alcohol, reading, exercise, etc.), nutrition, socioeconomic status (education, parental education, especially maternal education, and income, etc.), and other factors. Thus, we need to combine MRI with other nature and nurture data to better understand individual variability in neurocognition (Kessler et al., 2020; Skotting et al., 2021; Bolduc et al., 2018; Asschenfeldt et al., 2020; Oster et al., 2017; Savory et al., 2020; Derridj et al., 2021). There are technical challenges for (i) how to best combine 3D MRIs with 1D non-MRI features (Huang et al., 2020); (ii) how to identify the best subset of variables that optimally estimate neurocognitive abilities (Guyon and Elisseeff, 2003; Li et al., 2017); and (iii) how to eventually quantify and separate the contribution of nature vs. nurture. To unravel the contributions of nature (genetics) and nurture (environment and lifestyle) in explaining individual differences in neurocognition, combining MRI data with non-MRI features is essential. Below are some strategies to address the technical challenges identified,

#### 10.4.1 Combining 3D MRIs with 1D non-MRI features

Integrating complex 3D MRI data with 1D non-MRI variables (e.g., demographics, lifestyle factors) requires advanced multimodal data fusion techniques. Approaches such as multi-kernel learning or tensor decomposition can effectively handle multi-dimensional data by capturing the different relationships between brain structure and non-imaging variables. Additionally, the use of hybrid deep learning models, where CNNs process MRI data and other layers handle non-imaging data, can jointly model neuroimaging and non-neuroimaging information to improve predictions of neurocognition.

#### 10.4.2 Feature selection for multimodal data

Identifying the most informative features from both MRI and non-MRI data is key to understanding neurocognitive variability. Feature selection techniques like elastic net, recursive feature elimination, or random forest feature importance can be applied to both imaging and non-imaging datasets to extract the optimal subset of variables. Dimensionality reduction methods, such as PCA or CCA, can also reduce the complexity of large datasets, ensuring that the most relevant features are retained while minimizing redundancy.

#### 10.4.3 Quantifying and separating contributions of nature and nurture

The relative contributions of genetics (nature) and environmental factors (nurture) can be assessed using advanced statistical and machine learning methods. One approach is to use structural equation modeling (SEM) (Burnette and Williams, 2005) or twin studies to estimate heritability and disentangle genetic vs. environmental influences. In addition, techniques like partial least squares regression (PLSR) or mixed-effects models can help quantify how much variability in neurocognitive abilities is explained by MRI-derived brain features (reflecting biological aspects) and non-MRI features (reflecting environmental and lifestyle factors). These models allow researchers to separate and estimate the contribution of nature vs. nurture to cognitive outcomes.

#### 10.4.4 Causal inference and genetic data integration

To further separate the effects of nature and nurture, causal inference methods, such as Mendelian randomization (Sanderson et al., 2022) or propensity score matching, can be employed. These approaches enable the identification of causal relationships between genetic markers (e.g., polygenic scores) and neurocognitive abilities while controlling for confounding factors such as socioeconomic status or lifestyle choices. Integrating genetic data (nature) with MRI and environmental data (nurture) provides a more comprehensive view of how both domains influence brain development and cognitive outcomes.

### 10.5 Merging datasets

Artificial intelligence requires a large training dataset, which, for brain MRI, means 1,000 or more subjects (Smith and Nichols, 2018). Recent studies have combined public or private datasets to form a large database of thousands or even tens of thousands of brain MRIs, for age prediction (He et al., 2021a,b), quantification of normal brain development (He et al., 2018), genotype-phenotype mapping (Brookes and Robinson, 2015), and other tasks. We have found at least 38 public datasets with a total of about 35,000 unique individuals with both brain MRIs and neurocognitive/intelligence test scores (Table 2). Challenges arise, however, for (a) multi-site, multi-scanner, multi-protocol MRI harmonization; (b) dealing with different types or versions of neurocognitive tests as used in different datasets; (c) tackling uncertainties in the test scores for neurocognition/intelligence; and (d) coping with incompleteness or inconsistency in other variables (demographics, socioeconomic status, genetics, environment, etc.) across datasets. To tackle these challenges, the following steps can be taken:

**Table 2:** We have found at least 38 public datasets with a total of about 35,000 unique individuals with both brain MRIs and neurocognitive/intelligence test scores.

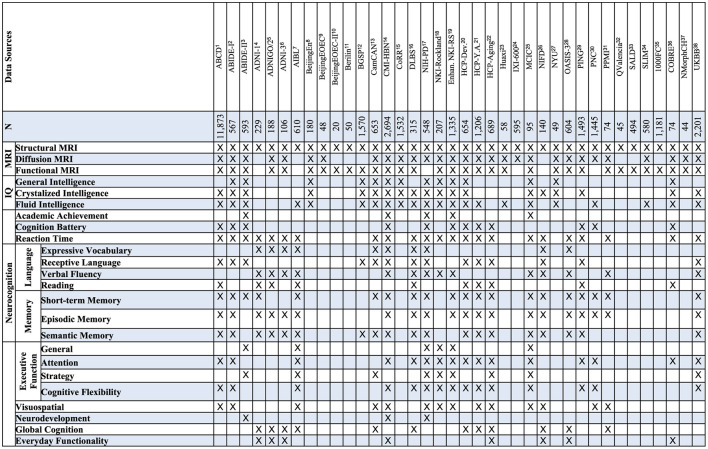

^1^ https://abcdstudy.org/

^2^ https://fcon_1000.projects.nitrc.org/indi/abide/abide_I.html

^3^ https://fcon_1000.projects.nitrc.org/indi/abide/abide_II.html

^4^ https://adni.loni.usc.edu/data-samples/adni-data/

^5^ https://adni.loni.usc.edu/data-samples/adni-data/

^6^ https://adni.loni.usc.edu/data-samples/adni-data/

^7^ https://aibl.csiro.au/adni/index.html

^8^ http://fcon_1000.projects.nitrc.org/indi/retro/BeijingEnhanced.html

^9^ http://fcon_1000.projects.nitrc.org/indi/retro/BeijingEOEC.html

^10^ http://rfmri.org/BeijingEOEC2_Raw

^11^ http://fcon_1000.projects.nitrc.org/indi/pro/Berlin.html

^12^ https://dataverse.harvard.edu/dataset.xhtml?persistentId=10.7910/DVN/25833

^13^ https://camcan-archive.mrc-cbu.cam.ac.uk/dataaccess/

^14^ https://fcon_1000.projects.nitrc.org/indi/cmi_healthy_brain_network/

^15^ https://fcon_1000.projects.nitrc.org/indi/CoRR/html/concept.html

^16^ https://fcon_1000.projects.nitrc.org/indi/retro/dlbs.html

^17^ https://nda.nih.gov/edit_collection.html?id=1151

^18^ https://fcon_1000.projects.nitrc.org/indi/pro/nki.html#LastRel

^19^ https://fcon_1000.projects.nitrc.org/indi/enhanced/index.html

^20^ https://www.humanconnectome.org/study/hcp-lifespan-development

^21^ https://www.humanconnectome.org/study/hcp-young-adult

^22^ https://www.humanconnectome.org/study/hcp-lifespan-aging

^23^ https://fcon_1000.projects.nitrc.org/indi/pro/wchsu_li_index.html

^24^ https://brain-development.org/ixi-dataset/

^25^ http://schizconnect.org/

^26^ https://memory.ucsf.edu/research-trials/research/allftd

^27^ https://fcon_1000.projects.nitrc.org/indi/pro/nyu.html

^28^ https://sites.wustl.edu/oasisbrains/

^29^ https://nda.nih.gov/study.html?id=477

^30^ https://www.ncbi.nlm.nih.gov/projects/gap/cgi-bin/study.cgi?study_id=phs000607.v3.p2

^31^ https://www.ppmi-info.org/

^32^ https://fcon_1000.projects.nitrc.org/indi/pro/Quiron-Valencia.html

^33^ https://fcon_1000.projects.nitrc.org/indi/retro/sald.html

^34^ https://fcon_1000.projects.nitrc.org/indi/retro/southwestuni_qiu_index.html

^35^ https://fcon_1000.projects.nitrc.org/fcpClassic/FcpTable.html

^36^ http://schizconnect.org/

^37^ http://schizconnect.org/

^38^https://www.ukbiobank.ac.uk/.

#### 10.5.1 Multi-site, multi-scanner, multi-protocol MRI harmonization

Harmonizing MRI data across multiple sites and scanners is a critical challenge due to differences in hardware and scanning protocols. One effective approach is to use statistical harmonization techniques like ComBat (Wang et al., 2017), which adjusts for scanner effects while preserving biological variability. Additionally, deep learning-based domain adaptation methods [review (Farahani et al., 2021)] can help normalize data across different sites without needing specific hand-engineered correction parameters.

#### 10.5.2 Handling different types or versions of neurocognitive test

Standardizing cognitive assessments across datasets is difficult due to varying test versions and scoring methods. One strategy is to apply crosswalk methodologies (Pritchard et al., 2024) that map scores from different versions to a common scale, allowing for more consistent comparisons across datasets. Furthermore, latent variable modeling can help harmonize cognitive measures by identifying common cognitive constructs despite differences in test versions.

#### 10.5.3 Dealing with uncertainties in test scores

Neurocognitive test scores may be subject to uncertainties due to testing conditions, examiner bias, or random variation. Bayesian modeling approaches provide a robust framework to incorporate uncertainty by generating probability distributions for test scores rather than relying on single-point estimates. These models allow for more flexible interpretations and uncertainty quantification in analyses.

#### 10.5.4 Coping with incompleteness or inconsistency in other variables

Missing data is a common issue in multi-dataset studies, especially regarding demographic, socioeconomic, genetic, or environmental variables. Advanced imputation techniques, such as multiple imputation by chained equations (MICE) (White et al., 2011) or machine learning-based imputation methods like k-nearest neighbors (KNN) or random forests, can help handle missing data effectively. Additionally, sensitivity analyses should be performed to assess how missing data influences the results, ensuring robustness across different datasets.

### 10.6 Evaluation of the present vs. prediction of the future

Predicting future neurocognitive outcomes and intelligence level is more difficult but is as important, if not more, than evaluating the current status. Early prediction of later-life neurocognitive outcomes will create a precious time window for early intervention (Liamlahi and Latal, 2019; Urschel et al., 2018). It will identify high-risk patients for targeted intervention, avoiding unnecessary interventions for patients at low risk for future neurocognitive impairments (Sterling et al., 2021). Both the early and the targeted interventions are key unmet needs in clinical trials that aim to improve patients' long-term neurocognitive outcomes (Urschel et al., 2018; Calderon and Bellinger, 2015). For the last three decades, there have been many studies that used medical imaging (e.g., MRI) and computer-aided mathematical models (e.g., multivariate analysis, machine learning, deep learning, etc.) to identify neurocognitive impairments in patients with various diseases, e.g., traumatic brain injuries (Cole J. H. et al., 2015), schizophrenia (Cole et al., 2018), Alzheimer's Disease (Franke et al., 2010), and diabetes (Franke et al., 2013). Yet, predicting normal and abnormal neurocognitive development trajectories remains a largely unanswered question. The prediction of future neurocognitive outcomes and intelligence levels is critical for enabling early intervention and improving long-term neurocognitive health. Below are some strategies to address the challenges of predicting future neurocognitive development:

#### 10.6.1 Longitudinal data and time series modeling

Predicting future neurocognitive outcomes requires longitudinal datasets that track individuals over time. Machine learning approaches that are specifically designed for time series data, such as recurrent neural networks (RNNs) or their variants like long short-term memory (LSTM) networks, can be used to model developmental trajectories. These methods account for temporal dependencies and can predict future cognitive states based on patterns in past data. Integrating longitudinal imaging and neurocognitive test data will improve our ability to forecast later-life outcomes and identify early deviations from normal trajectories.

#### 10.6.2 Transfer learning for early prediction

Transfer learning offers an approach for improving predictions in cases with limited early-life data by leveraging models trained on large datasets of older populations. Pre-trained models from adult neuroimaging studies can be fine-tuned using pediatric data to predict later-life cognitive outcomes. This method reduces the need for extensive early-life data and provides a more efficient framework for predicting long-term neurocognitive outcomes from early MRI scans and neurodevelopmental profiles.

#### 10.6.3 Risk stratification for targeted interventions

Early prediction models can be designed to stratify patients into risk categories (e.g., high-risk vs. low-risk for future neurocognitive impairments). Techniques like survival analysis or Cox proportional hazards models can assess the probability of neurocognitive decline over time. More advanced models, such as gradient-boosted decision trees or deep learning classifiers, can also identify patients at higher risk for cognitive impairments based on baseline MRI and other clinical factors. These risk stratification models will guide the development of personalized intervention strategies, allowing for targeted prevention in high-risk groups while avoiding unnecessary treatments in low-risk individuals.

#### 10.6.4 Multimodal data integration for comprehensive predictions

To accurately predict future neurocognitive outcomes, it is crucial to incorporate not only MRI data but also genetic, behavioral, environmental, and lifestyle factors. Multimodal data integration using machine learning models, such as multimodal neural networks or ensemble approaches, can combine different types of data (e.g., neuroimaging, genetics, and clinical profiles) to provide a more holistic view of an individual's cognitive trajectory. This integrative approach can lead to more precise predictions of normal and abnormal neurocognitive development across the lifespan.

#### 10.6.5 Early biomarker identification and personalized trajectories

Identifying early biomarkers of cognitive decline or abnormal neurodevelopment is key to predicting future outcomes. Advanced neuroimaging methods, including dMRI and fMRI, combined with feature selection algorithms, can help pinpoint critical brain regions or connectivity patterns that are predictive of long-term cognitive outcomes. Personalized prediction models, which account for an individual's unique brain characteristics, genetic predispositions, and environmental exposures, will further refine future outcome predictions and allow for more tailored early interventions.

### 10.7 Linking healthy and diseased

Do machine intelligence models that predict human intelligence in normal controls help us predict abnormal neurocognitive outcomes in diseased populations? Do neurocognitive outcome prediction models share similar MRI and non-MRI features across diseases? Current studies of diseased populations often focus on one specific disease at a time. Linking healthy and diseased, or merging data across diseases, may offer new insight for the common support of normal and abnormal neurocognitive development.

## 11 Conclusion

In this paper, we reviewed different MRI studies that inferred neurocognitive or human intelligence. While existing reviews are often on specific disease populations, our review focuses primarily on healthy subjects but has included various disease-specific MRI findings. We observed several trends in this research direction: population-level association studies are transitioning to individual-level machine learning predictions, integrating MRI with rich non-MRI information, and bigger sample sizes (thousands or tens of thousands) by merging datasets are fast increasing compared to small sample size studies (dozens to hundreds) from a single dataset. Despite growing efforts and expanding knowledge, the decades-long topic of artificial intelligence inferring human intelligence remains little understood in general. Opportunities exist with the rise of big data and AI, but several major neuroscientific and data science challenges call for further investigations.
