# Supplementary material for: Inferring neurocognition using artificial intelligence on brain MRIs
Source: Front Neuroimaging. 2024 Nov 27;3:1455436. doi: 10.3389/fnimg.2024.1455436 (PMC11631947; doi:10.3389/fnimg.2024.1455436)
Supplement: Supplementary file 1 [file Image_1.pdf]

Supplementary Material

1. Structural Brain MRI Pre-processing and Feature Extraction

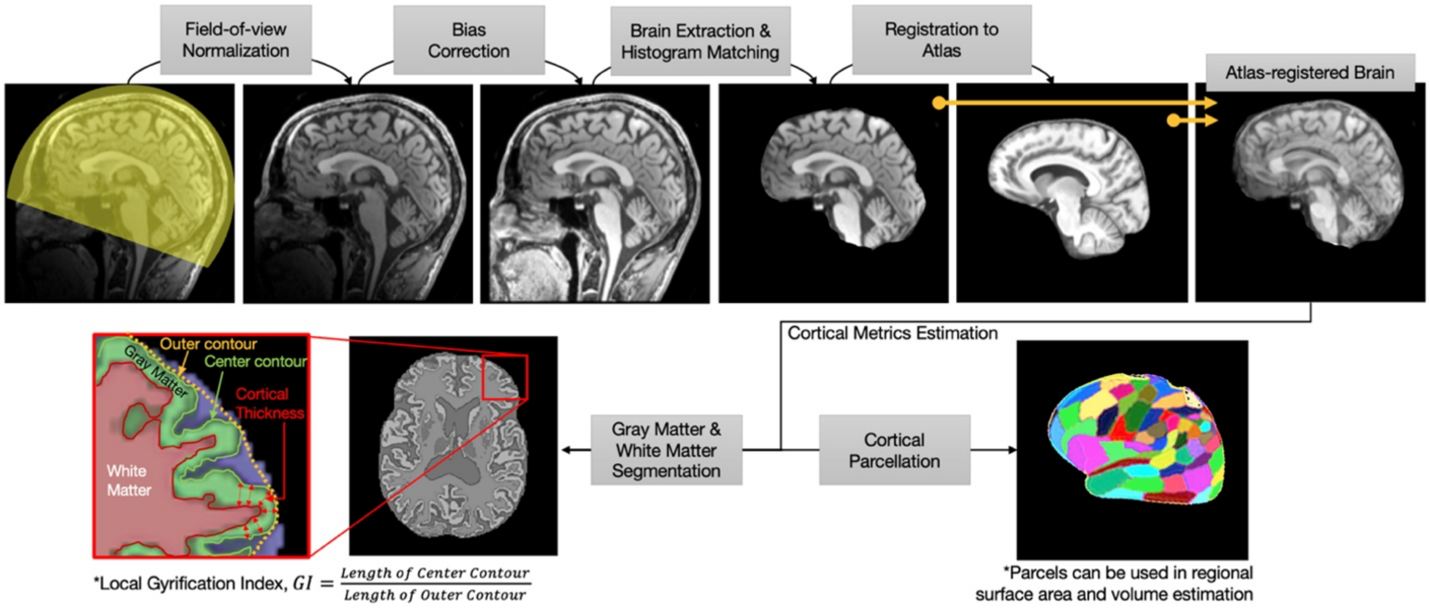

Supplementary Figure 1. *Typical structural brain MRI pre-processing and feature extraction pipeline.*

Supplementary Table 1*. Summary of sMRI studies correlating regional brain volumes with neurocognition/intelligence. Acronyms- ANCOVA: Analysis of Covariance, ABCD: Adolescent Brain Cognitive Development, NKI: Nathan S. Kline Institute for Psychiatric Research, NIH-TCB: NIH toolbox of neurocognitive battery, PCA: Principal Component Analysis, LASSO: Least Absolute Shrinkage and Selection Operator, SVM: Support Vector Machine, SVR: Support Vector Regression, RF: regression/random Forest, LR: linear regression, RR: ridge regression, MLP: multi-layer perceptron, CNN: Convolutional Neural Network, ROI: Region of Interest, KNN: K-Nearest Neighbors, MSE: Mean Square Error, RMSE: Root MSE, WASI: Weschler Abbreviated Scale of Intelligence, WISC: Wechsler Intelligence Scale for Children, WAIS: Wechsler Adult Intelligence Scale, FSIQ: Full-scale Intelligent Quotient, BOMAT: Bochum Matrices Test, T1-w: T1-weighted MRI, T2-w: T2-weighted MRI, P-FGR: Pre-term Fetal Growth Restricted, PT-AGA: Pre-term Appropriate Gestational Age, T-AGA: Term AGA, DTI: Diffusion Tensor Imaging, ICV: intracranial volume, WM: white matter, GM: gray matter, CSF: cerebrospinal fluid. Probable BAs are not specified for either the left or right hemisphere.*

Serial	Study	Year	N	Age (years)	Dataset	MRI type	MRI features	Regions	Probable BAs	IQ/Neuro. Test	Normal/ Abnormal	Method	Correlation/ Finding
1	Nave et al.[49]	2018	13,608	48-69	UK Biobank	T1-w	Total Brain Volume (TBV)	GM, WM, CSF	Not Specified	Not Specified	Normal	Least Square Regression	Association between the TBV and fluid intelligence, r = 0.19
2	Saha et al.[63]	2021	7,709	9-10	ABCD	T1-w	CNN learned features and volumes of manually identified brain regions	GM regions of left/right hippocampus, parahippocampal gyrus, thalamus, precentral gyrus, and caudate nucleus; WM region of the pons.	34, 4	NIH-TCB	Normal	CNN and MLP	Correlation between the actual and predicted $gF = 0.1$ ($p < 0.05$)

3	Hilger et al.[64]	2020	380	18-60	NKI-Rockland-Enhanced	T1-w	GM volume per voxel	Frontoparietal network, default mode network, Dorsal attention network, and cerebellum	38, 25, 23, 31, 4, 17, 18, 19, 8, 7, 6, 9	WASI	Normal	PCA+Linear SVR	MSE and correlation between the actual and estimated FSIQ is 320 (p = 0.279) and 0.11, respectively (for true residual FSIQ in the range of [39, 136])
4	Chiang et al.[53]	2019	8,669	9-10	ABCD	T1-w	Total volume, mean signal intensity, and entropy	Visual, frontoparietal, somatosensory, motor, default mode network, and cingulo-opercular network.	6, 8, 9, 22, 41, 42, 17, 18, 19, 1, 2, 3, 5, 7, 4	NIH-TCB	Normal	CNN, and LASSO	Mean Square Error *(gF)* = 95.38 (for true residual *gF* in the range of [-40, 30])
5	Shrivastava et al.[54]	2019	8,669	9-10	ABCD	T1-w	Volume, mean intensity, and count of GM voxels	Gyrus rectus, hippocampus, inferior frontal gyrus, middle frontal gyrus, postcentral gyrus, precentral gyrus, precuneus, superior frontal gyrus and supramarginal gyrus.	11, 44, 45, 47, 4, 1, 2, 3, 10, 12, 40	NIH-TCB	Normal	CNN, SVR, RF, gradient boosting, and XGBoost	Mean Square Error *(gF)* = 93.68 (for true residual *gF* in the range of [-40, 30])
6	Ren et al.[55]	2019	8,669	9-10	ABCD	T1-w	ROI volumes	GM	11, 44, 45, 47, 4, 1, 2, 3, 10, 12, 40	NIH-TCB	Normal	Bagging and boosting of LR, RR, RF, envelope-based reduced-rank regression, LASSO, Elastic-Net regressor, and KNN	Mean Square Error *(gF)* = 92.99 (for true residual *gF* in the range of [-40, 30])
7	Tamez-Pena et al.[56]	2019	8,669	9-10	ABCD	T1-w	ROI volumes	GM, WM, CSF, and cerebellum	11, 44, 45, 47, 4, 1, 2, 3, 10, 12, 40	NIH-TCB	Normal	Ensemble of SVM, RF, and bootstrapped step wise model selection	Mean Square Error *(gF)* = 100.89 (for true residual *gF* in the range of [-40, 30])
8	Brueggeman et al.[57]	2019	8,669	9-10	ABCD	T1-w	122 ROI volumes	GM, WM, CSF	11, 44, 45, 47, 4, 1, 2, 3, 10, 12, 40	NIH-TCB	Normal	RF	Mean Square Error *(gF)* = 92.49 (for true residual *gF* in the range of [-40, 30])
9	Mihalik et al.[58]	2019	8,669	9-10	ABCD	T1-w	Voxel intensities and probabilistic tissue-type labels	GM, WM	11, 44, 45, 47, 4, 1, 2, 3, 10, 12, 40	NIH-TCB	Normal	Kernel ridge regressor	Mean Square Error *(gF)* = 92.13 (for true residual *gF* in the range of [-40, 30])
10	Ranjbar et al.[59]	2019	8,669	9-10	ABCD	T1-w	122 ROI volumes	GM, WM, CSF	11, 44, 45, 47, 4, 1, 2, 3, 10, 12, 40	NIH-TCB	Normal	CNN and RF	Mean Square Error *(gF)* = 93.64 (for true residual *gF* in the range of [-40, 30])
11	Wlaszczyk et al.[60]	2019	8,669	9-10	ABCD	T1-w	ROI volumes, signal intensity, anterior and posterior cross-sectional area from corpus callosum	GM and corpus callosum	11, 44, 45, 47, 4, 1, 2, 3, 10, 12, 40	NIH-TCB	Normal	RF	Mean Square Error *(gF)* = 92.93 (for true residual *gF* in the range of [-40, 30])
12	Zhang-James et al.[47]	2019	8,669	9-10	ABCD	T1-w	122 ROI volumes	GM, WM, CSF	11, 44, 45, 47, 4, 1, 2, 3, 10, 12, 40	NIH-TCB	Normal	Nu SVM	Mean Square Error *(gF)* = 95.63 (for true residual *gF* in the range of [-40, 30])
13	Kao et al.[61]	2019	8,669	9-10	ABCD	T1-w	122 ROI volumes	GM, WM, CSF	11, 44, 45, 47, 4, 1, 2, 3, 10, 12, 40	NIH-TCB	Normal	StackNet consisting of random forest, random tree, ridge regressor, and gradient boosting	Mean Square Error *(gF)* = 94.25 (for true residual *gF* in the range of [-40, 30])
14	Li et al.[62]	2019	8,669	9-10	ABCD	T1-w	ROI volumes, the number of detected surface holes, the globus pallidus volume, the mean curvatures of precentral gyrus, postcentral gyrus, and banks of Superior Temporal Sulcus	Right posterior cingulate gyrus, left caudate nucleus, entorhinal white matter, globus pallidus, precentral gyrus, postcentral gyrus, and superior temporal sulcus	23, 31, 28, 4, 1, 2, 3, 22	NIH-TCB	Normal	BlockPC-XGBoost	Mean Square Error *(gF)* = 93.16 (for true residual *gF* in the range of [-40, 30])
15	Morsing et al.[51]	2018	74	7-8	Skane University Hospital in Lund, Sweden	T1-w	ROI volumes	ICV, GM, WM, CSF, and thalamus.	Not Specified	WISC-III	P-FGR, PT-AGA, and T-AGA	Chi-square and ANOVA	The mean (SD) FSIQ was 80 (17) in the PT-FGR group and 103 (12) in the PT-AGA group

16	Ogawa et al.[52]	2018	232	21-69	Advanced Telecommunication Research Institute International, Kyoto	T1-w	GM volume	Right insula, right middle cingulate cortex/precuneus	13, 14, 16, 4	Insight test battery (ITB)	Normal	Linear regression	ITB score was positively correlated with the GM volumes in the mentioned region (p < 0.001)
17	Paul et al.[50]	2016	211	18-44	University of Illinois Urbana-Champaign	T2-w	Volume fractions across tissue types	GM, WM, CSF	23, 31	BOMAT, Number Series, and Letter Set	Normal	Bivariate correlation	GM volume is found positively correlated with quantitative reasoning (r = 0.26; p < 0.01) and working memory (r = 0.21; p < 0.01), and *gF* (r = 0.16; p < 0.01)
18	Grazioplene et al.[67]	2015	517	18-40	University of Minnesota, University of New Mexico in Albuquerque, Yale University	T1-w MPRAGE	Caudate volume	Caudate nucleus	Not Specified	WAIS-III, WAIS-IV, WASI	Normal	Linear regression	Regression of IQ onto bilateral caudate volume indicated a significant positive correlation between caudate volume and FSIQ (r = 0.24; p = 0.01)

Supplementary Table 2. *Summary of sMRI studies correlating cortical surface metrics with neurocognition/intelligence. Acronyms- ABCD: Adolescent Brain Cognitive Development, NIH-TCB: NIH toolbox of neurocognitive battery, LASSO: Least Absolute Shrinkage and Selection Operator, SVM: Support Vector Machine, SVR: Support Vector Regression, CNN: Convolutional Neural Network, ROI: Region of Interest, KNN: K-Nearest Neighbors, MSEL: Mullen Scale of Early Learning, PMAT: Penn Progressive Matrices, RIAS: Reynolds Intellectual Assessment Scales, RPM: Raven's Advanced Progressive Matrices Set, GM: Gross Motor, VR: Visual Reception, FM: Fine Motor, RL: Receptive Language, EL: Expressive Language, ELC: Early Learning Composite, CFT: Cluster Forming Threshold, RMSE: Root Mean Square Error, WISC: Wechsler Intelligence Scale for Children, WAIS: Wechsler Adult Intelligence Scale, ABIDE: Autism Brain Imaging Data Exchange, BOLD: Blood-oxygenation Level-dependent, T1-w: T1-weighted MRI, T2-w: T2-weighted MRI, DWI: Diffusion-weighted Imaging. Probable BAs are not specified for either the left or right hemisphere.*

Serial	Study	Year	N	Age (years)	Dataset	MRI type	MRI features	Regions	Probable BAs	IQ/Neuro. Test	Normal/ Abnormal	Method	Correlation/ Finding
1	Li et al.[75]	2020	68	8	Arkansas Children's Nutrition Center	T1-w	Gray matter volume, surface area, and cortical thickness	Orbitofrontal gyrus, transverse temporal gyri, left superior temporal gyrus, and right anterior cingulate gyrus	11, 12, 41, 42, 22, 24, 32, 33	RIAS	Normal	Spearman's correlation	RIAS scores showed significant correlations (r = [0.38-0.44], p = [0.005-0.046]) with cortical metrices
2	Tadayon et al.[77]	2020	740	21-35	HCP	T1-w	Cortical thickness, cortical surface area, and cortical gyrification	Superior parietal, left supramarginal, left caudal middle frontal, left pars-opercularis, left inferior temporal, right inferior and middle temporal, right medial orbitofrontal, and right rostral middle frontal regions	7, 40, 22, 44, 20, 21, 11, 12, 10	PMAT and NIH-TCB	Normal	Linear regression	Correlation between the local gyrification, and surface area with *gF* and *gC* are 0.29 and 0.22 (p < 0.001), 0.28 and 0.28 (p < 0.001), respectively
3	Oxtoby et al.[82]	2019	8,669	9-10	ABCD	T1-w	Cortical morphology as graph	A structural co-variance network graph considers small cortical regions (3 voxels cubed) as nodes, and structural similarity (morphology) between nodes as edges.	11, 44, 45, 47, 4, 1, 2, 3, 10, 12, 40	NIH-TCB	Normal	Event-based model of progression, and SVR	Mean Square Error *(gF)* = 93.83 (for true residual *gF* in the range of [-40, 30])
4	Rebsamen et al.[83]	2019	8,669	9-10	ABCD	T1-w	Subcortical volumes, cortical thicknesses, curvatures, and surface areas	Middle temporal gyrus, superior temporal gyrus	21, 22	NIH-TCB	Normal	SVR	Mean Square Error *(gF)* = 93.03 (for true residual *gF* in the range of [-40, 30])
5	Valverde et al.[84]	2019	8,669	9-10	ABCD	T1-w	122 ROI volumes in the gray matter, white matter, and cerebrospinal fluid, 78 contrast and 78 cortical thickness measures, gender, age, and scanner manufacturer	Gray matter, white matter, and cerebrospinal fluid	Not Specified	NIH-TCB	Normal	Fully connected neural network	Mean Square Error *(gF)* = 94.02 (for true residual *gF* in the range of [-40, 30])
6	Pölsterl et al.[85]	2019	8,669	9-10	ABCD	T1-w	Cortical thickness and volumes of 122 ROIs in the gray matter, white matter, and cerebrospinal fluid	Left/right parahippocampal gyrus, pons white matter, hippocampus,	34, 23, 31, 19, 10, 40,	NIH-TCB	Normal	An ensemble of gradient boosted	Mean Square Error *(gF)* = 94.25 (for true residual *gF* in

								posterior cingulate gyrus, cuneus, left lingual gyrus, left middle frontal gyrus, supramarginal gyrus, right fusiform gyrus, superior temporal gyrus, right anterior cingulate gyrus, etc.	37, 22, 24, 32, 33			trees, and a linear ridge regressor.	the range of [-40, 30])
7	Pölsterl et al.[86]	2019	8,669	9-10	ABCD	T1-w	Cortical thickness and volumes of 122 ROIs in the gray matter, white matter, and cerebrospinal fluid	Left/right parahippocampal gyrus, pons white matter, hippocampus, posterior cingulate gyrus, cuneus, left lingual gyrus, left middle frontal gyrus, supramarginal gyrus, right fusiform gyrus, superior temporal gyrus, right anterior cingulate gyrus, etc.	34, 23, 31, 19, 10, 40, 37, 22, 24, 32, 33	NIH-TCB	Normal	AutoML ensembles of 14 classifiers	Mean Square Error *(gF)* = 94.25 (for true residual *gF* in the range of [-40, 30])
8	Guerdan et al.[87]	2019	8,669	9-10	ABCD	T1-w	Volume, elongation, surface area, roundness, and flatness of grey matter ROIs.	Gray matter, white matter, and cerebrospinal fluid	Not Specified	NIH-TCB	Normal	LASSO, ridge regressor, SVR, gradient boosting, and AdaBoost regressors.	Mean Square Error *(gF)* = 94.48 (for true residual *gF* in the range of [-40, 30])
9	Girault et al.[78]	2019	487	1-2	University of North Carolina (UNC) Chapel Hill Early Brain Developm ent Study	T1-w, T2-w	Cortical thickness, and surface area	Bilateral superior frontal and middle frontal gyri, right medial superior frontal gyrus, right occipital superior gyrus, bilateral superior parietal cortices, left primary motor cortex, bilateral anterior cingulate and precuneus, and right superior and middle temporal cortices areas	10, 19, 7, 4, 24, 32, 33, 22	GM, VR, FM, RL, EL, and ELC (MSEL)	Normal	Pearson correlation, Linear mixed effect model	Correlations between average cortical thickness at age 1 and GM, FM, EL, and RL scores at age 1 ($r = 0.137$, $p = 0.025$; $r = 0.186$, $p = 0.002$; $r = 0.147$, $p = 0.016$; $r = 0.120$, $p = 0.049$, respectively),
10	Adeli et al.[88]	2019	24	0-4	UNC Chapel Hill Early Brain Developm ent Study	T1-w, T2-w, DWI	Cortical thickness, mean curvature, local gyrification index, vertex area, vertex volume, sulcal depth in string distance, and sulcal depth in Euclidean distance	Parcellation of the cerebral cortex into 70 anatomically meaningful ROIs	Not Specified	VR, FM, RL, EL, and ELC (MSEL)	Normal	Multi-task multi-linear regression	Average RMSE between the predicted and actual VR, FM, RL, and EL scores is 0.18.
11	Zhang et al.[89]	2018	23	0-4	UNC Chapel Hill Early Brain Developm ent Study	T1-w, T2-w	Cortical thickness, mean curvature, local gyrification index, vertex area, vertex volume, sulcal depth in string distance, and sulcal depth in Euclidean distance	Parcellation of the cerebral cortex into 70 anatomically meaningful ROIs	Not Specified	VR, FM, RL, EL, and ELC (MSEL)	Normal	Multi-task multi-linear regression	Average RMSE between the predicted and actual VR, FM, RL, and EL score is 0.158.
12	Zhang et al.[90]	2020	23	0-4	UNC Chapel Hill Early Brain Developm ent Study	T1-w, T2-w	Cortical thickness, mean curvature, local gyrification index, vertex area, vertex volume, sulcal depth in string distance, and sulcal depth in Euclidean distance	Parcellation of the cerebral cortex into 70 anatomically meaningful ROIs	Not Specified	VR, FM, RL, EL, and ELC (MSEL)	Normal	CNN	Average RMSE between the predicted and actual VR, FM, RL, and EL scores is 0.067.
13	Cheng et al.[91]	2022	23	0-4	UNC Chapel Hill Early Brain Developm ent Study	T1-w, T2-w	Cortical thickness, mean curvature, local gyrification index, vertex area, vertex volume, sulcal depth in string distance, and sulcal depth in Euclidean distance	Parcellation of the cerebral cortex into 70 anatomically meaningful ROIs	10, 11, 17, 19, 22, 23, 28, 31, 34, 37, 39, 40, 45, 47	VR, FM, RL, EL, and ELC (MSEL)	Normal	Cortical Feature-based Path Signature Neural Network (CF-PSNet)	Average RMSE between the predicted and actual VR, FM, RL, and EL scores is 0.023.
14	Cheng et al.[92]	2023	318	0-3	UNC/UM N Baby Connecto me Project	T1-w, rs-fMRI	Cortical thickness, surface area, cortical volume, sulcal depth, mean curvature, and average convexity	432 cortical ROIs per hemisphere	Not Specified	VR, FM, RL, and EL (MSEL)	Normal	Cortical surface-based multimodal learning framework (CSML)	Average RMSE between the predicted and actual VR, FM, RL, and EL scores is 0.0915.
15	Bajaj et al.[76]	2018	56	18-45	McLean Hospital and Partners Healthcare , and the U.S. Army	T1-w	Cortical thickness, cortical surface area, cortical volume, and cortical gyrification	Posterior frontal, superior and inferior parietal lobes, left insula, and inferior frontal gyrus	7, 39, 40, 13, 14, 16, 44, 45, 47	WASI-II	Normal	Generalized linear model	Significant positive relationships between thicker cortex and higher IQ at a liberal CFT of $p < 0.05$ as well as at a strict CFT of

					Human Research Protections Office							$p < 0.01$ is observed.
16	Wang et al.[93]	2015	164	6-15	ABIDE	T1-w	Cortical thickness, surface area, sulcal depth, curvature	Bilateral transverse temporal gyri, bilateral thalamus, left parahippocampal gyrus, left hippocampus, right opercular part of inferior frontal gyrus, left anterior cingulate gyrus, right amygdala, left lingual gyrus, left superior parietal lobule, right inferior parietal lobule, left angular gyrus, left paracentral lobule, and left caudate nucleus	41, 42, 34, 44, 45, 47, 32, 7, 40, 39, 1, 2, 3, 4	-	Normal	Multi/single kernel support vector regressor	Correlation between the actual and estimated IQ is 68.4%
17	Squeglia et al.[79]	2013	185	12-14	San Diego area public middle schools	T1-w	Cortical thickness	Left and right inferior parietal cortices, and left and right superior parietal cortices	39, 40, 7	WISC-III, WAIS-IV	Normal	Hierarchical linear regressions	For both males and females, thinner parietal association cortices corresponded with better neurocognitive functioning above and beyond age alone.
18	Yang et al.[80]	2013	78	17-27	Seoul National University, Catholic University of Korea	T1-w	Cortical thickness, surface area, sulcal depth and absolute mean curvature in 78 parcellated ROIs	Cerebral cortex	34, 35, 37	WAIS	Normal	Partial least square regression	Correlation between the Actual and predicted FSIQ is 30% ($p < 0.01$)
19	Choi et al.[81]	2008	225	20.9±2.9	Seoul National University, Catholic University of Korea	T1-w	The thickness of the gray matter of the cerebral cortex	Gray matter of cerebral cortex	38, 20, 21, 40	WASI, RPM-II	Normal	Multivariate regression model	*gC* is correlated to cortical thickness and *gF* is related to BOLD signals.

Supplementary Table 3. *Summary of sMRI study using brain morphometry in inferring/relating to human neurocognition and intelligence. Acronyms- COPD: Chronic Obstructive Pulmonary Disorder, OCD: Obsessive Compulsive Disorder, DD: Developmental Dyslexia, FA: fractional anisotropy, VBM: Voxels-based Morphometry, MDD: major Depressive Disorder, NCANDA: National Consortium on Alcohol and Neurodevelopment in Adolescence, WASI: Weschler Abbreviated Scale of Intelligence, WISC: Wechsler Intelligence Scale for Children, WAIS: Wechsler Adult Intelligence Scale, FSIQ: Full-scale Intelligent Quotient, VIQ: Verbal IQ, PIQ: Performance IQ, ANCOVA: Analysis of Covariance, T1-w: T1-weighted MRI, DTI: Diffusion Tensor Imaging. Probable BAs are not specified for either the left or right hemisphere.*

Serial	Study	Year	N	Age (years)	Dataset	MRI type	MRI features	Regions	Probable BAs	IQ/Neuro. Test	Normal/ Abnormal	Method	Correlation/ Finding
1	Hidese et al.[97]	2020	266	45.6±12.9	Volunteer data from Kodaira city, Tokyo	T1-w, DTI	Regional gray matter volumes in the VBM and the white matter FA values in the DTI	Left gyrus rectus and anterior cingulate gyrus, left posterior insula, left superior and middle frontal gyri	11, 24, 32, 33, 13, 14, 16, 10	WAIS-III	Normal	Pearson correlation	VIQ correlated positively with the specified brain regional volumes with *t* score = 4.94; $p < 0.005$.
2	McDermott et al.[98]	2019	623	5-25	National Institute of Mental Health Intramural Research Program	T1-w	Surface-based shape	Left inferior and middle temporal, left inferior parietal, and left medial frontal regions	20, 21, 39, 40, 25	WASI, WISC, WAIS	Normal	Linear mixed-effect model	Positive associations ($\beta > 100$; $p < 0.001$) between FSIQ and cortical anatomy is observed.

3	Ramsden et al.[99]	2011	33	14.1±1.0	Department of Psychological Sciences, Birkbeck College, University of London	T1-w	Changes in gray matter density	Motor speech area, and anterior cerebellum	4, 6	WISC, WAIS	Normal	Linear regression	Correlation between change in VIQ and change in grey matter density were 0.876 ($p < 0.01$) for high, 0.797 ($p < 0.05$) for average and 0.660 ($p < 0.05$) for low ability groups, respectively. For PIQ, correlation was 0.492 ($p > 0.05$) for high, 0.788 ($p < 0.05$) for average and 0.715 ($p < 0.01$) for low ability groups, respectively.

2. Diffusion MRI Pre-processing and Feature Extraction

Diffusion MR images are preprocessed to enhance data quality and accuracy. One important step is Eddy current correction, which corrects distortions caused by magnetic field inhomogeneities during imaging. This correction involves aligning the diffusion-weighted images to a reference image, often using methods like affine registration or geometric distortion correction algorithms. Additionally, preprocessing may include denoising to remove noise, motion correction to compensate for subject motion during scanning, and gradient distortion correction to address spatial distortions in gradient directions. These steps help improve the reliability and interpretability of diffusion MRI data. Diffusion tensor imaging (DTI) is a specific type of diffusion MRI technique that assumes a tensor solution to quantify the diffusion properties of water molecules within tissues. It is widely used to investigate the structural connectivity and organization of white matter tracts in the brain. DTI utilizes the diffusion of water along multiple directions to estimate a diffusion tensor, which describes the magnitude and directionality of water diffusion in each voxel of the image (see Supplementary Figure 2). There are several other advanced diffusion MRI techniques for tractography, such as Neurite Orientation Dispersion and Density Imaging (NODDI) and Diffusion Spectrum Imaging (DSI), which offer more detailed information about tissue microstructure compared to DTI. However, DTI is the most basic and most widely used technique. By analyzing the diffusion tensor, various measures can be derived, including fractional anisotropy (FA, which measures the directionality of water diffusion, ranging between 0 for completely isotropic diffusion in all directions and 1 for single-directional diffusion), mean diffusivity (MD, which measures the magnitude of water diffusion), axial diffusivity (AD), which measures the rate of diffusion of water molecules along the principal axis of diffusion, and radial diffusivity (RD), which measures the rate of diffusion of water molecules perpendicular to the principal axis of diffusion.

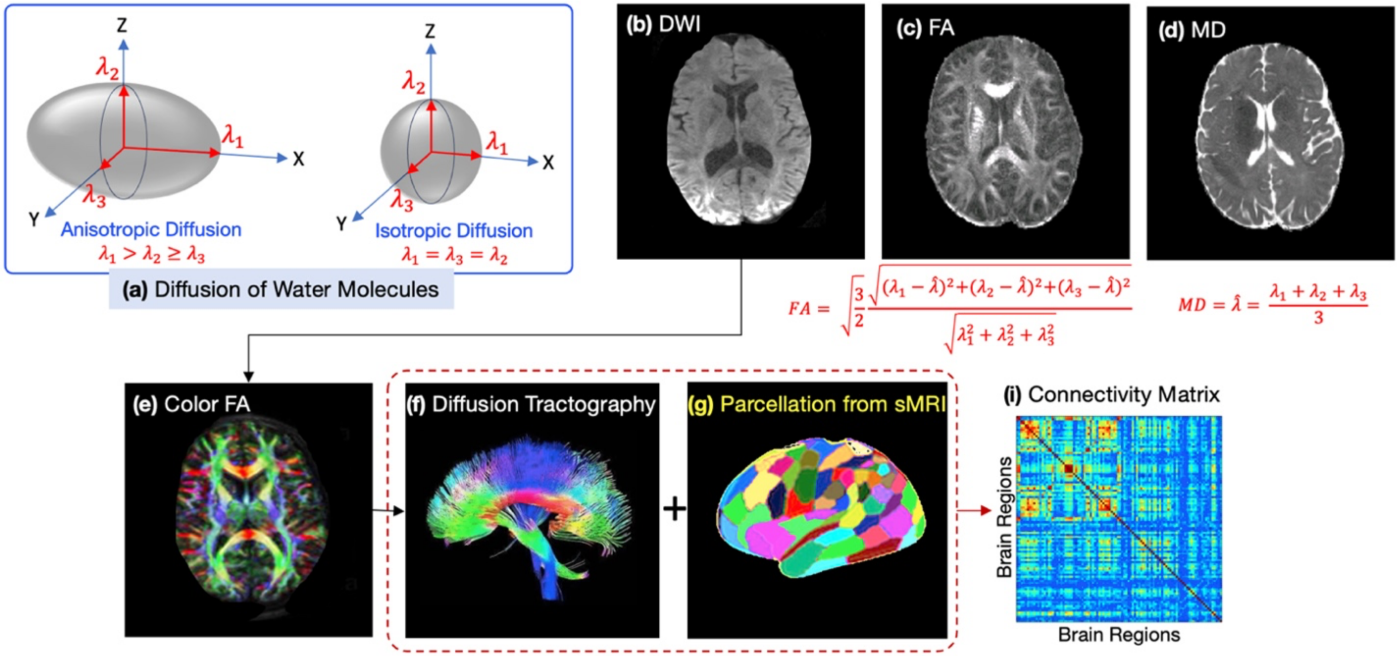

Supplementary Figure 2. *(a) Diffusion Imaging typically utilizes water diffusivity metrics, e.g., eigenvalues (λ) of a diffusion tensor. In isotropic diffusion, water molecules diffuse equally in all directions, i.e., $\lambda_1 = \lambda_3 = \lambda_2$, while anisotropic diffusion occurs when water molecules preferentially diffuse along certain directions rather than others, e.g., $\lambda_1 > \lambda_2 \geq \lambda_3$. (b) Typical Diffusion-weighted Image (DWI), (c) Fractional Anisotropy (FA), and (d) Mean Diffusivity (MD) encoding magnitude in black and white and with I color encoding the principal axis of the diffusion direction/map (Color FA). Diffusion Imaging utilizes water diffusivity metrics in the brain to perform (f) tractography, where fiber tracts connecting to (g) different parcels provide (i) structural connectivity information in the brain.*

Supplementary Table 4. *Summary of diffusion MRI (i.e., DWI and DTI) studies inferring human neurocognition and intelligence. Acronyms- FA: Fractional Anisotropy, MD: Mean Diffusivity, RD: Radial Diffusivity, BSID: Bayley Scales of Infant Development, MSEL: Mullen Scale of Early Learning, MMSE: Mini-Mental Status Examination, TBSS: tract-based spatial statistics, VR: Visual Reception, FM: Fine Motor, RL: Receptive Language, EL: Expressive Language, ELC: Early Learning Composite, RMSE: Root Mean Square Error, HCP: Human Connectome Project, WASI: Weschler Abbreviated Scale of Intelligence, WISC: Wechsler Intelligence Scale for Children, WAIS: Wechsler Adult Intelligence Scale, FSIQ: Full-scale Intelligent Quotient, PIQ: Performance IQ, VIQ: Verbal IQ, T1-w: T1-weighted MRI, T2-w: T2-weighted MRI, DWI: Diffusion-weighted Imaging, DTI: Diffusion Tensor Imaging. Probable BAs are not specified for either the left or right hemisphere.*

**Serial**	**Study**	**Year**	**N**	**Age (years)**	**Dataset**	**MRI type**	**MRI features**	**Regions**	**Probable BAs**	**IQ/Neuro. Test**	**Normal/ Abnormal**	**Method**	**Correlation/ Finding**
1	Malpas et al.[101]	2016	91	18-55	Nathan Kline Institute/Rockland Sample	DTI, fMRI	FA	42 Brodmann regions were specified in each hemisphere	1, 3, 4, 5, 6, 7, 8, 9, 11, 24, 25, 29, 32, 44, 45, 46, 47, 13, 22, 34, 35, 36, 38, 41, 42, 39, 40, 43, 17, 18	WASI	Normal	*t* statistic regression analysis	FA was positively correlated with FSIQ with r = 0.53 (95% CI 0.35–0.66).
2	Konrad et al.[111]	2012	30	22.8±1.5	Institute of Neuroradiology of the Johannes Gutenberg University Mainz, Germany	T1-w, DTI	FA, MD	Left-hemispheric Broca's area	44, 45, 22	Hamburg–Wechsler Intelligenztest (HAWIE-R) - equivalent to WAIS-R	Normal	Voxel-wise *t* statistic regression analysis, Pearson correlation	VIQ performance is negatively correlated to the FA in the mentioned regions (r = - 0.73; p < 0.001).

3	Feng et al.[110]	2019	38	0-2	Arkansas Children's Nutrition Center	DTI	FA	White matter tracts	Not Specified	BSID-III	Normal	Voxel-wise TBSS	Correlations between FA at 2 weeks of age and BSID subfields scores at 2 years of age are 0.35~0.48.
4	Casson et al.[112]	2014	45	30-60	Wayne State University	T1-w, SWI, DTI	FA-based dysarthria, pyramidal system dysfunction, extrapyramidal system dysfunction, and cerebellar dysfunction	Gray matter, white matter, and cerebrospinal fluid	Not Specified	MMSE	Normal/ abnormal	Chi-square test	The number of football-related concussions was associated with isolated neurocognitive abnormalities in 24% of population.
5	Lee et al.[113]	2017	535	0-2	UNC Chapel Hill Early Brain Development Study	DTI	Axial diffusivity (AD), radial diffusivity (RD), and FA	White matter	Not Specified	MSEL: ELC	Normal	Distance correlation	Correlation between AD, RD and FA with ELC are 0.13~0.20 ($p < 0.05$)
6	Zhang et al.[120]	2019	1076	-	HCP	DWI	Count of streamlines, connected surface area (CSA) and weighted CSA, mean and maximum values of FA and MD, cluster number, average length, and mean deviations from a template streamline	ROIs in the whole cortex	Not Specified	Raven's Progressive Matrices	Normal	Latent partial multi-view representation learning (multi-task learning)	Correlation between actual and estimated *gF* is 24.11% ($p < 0.001$).
7	Clayden et al.[106]	2012	59	8-16	Local Schools in London, UK	DTI	FA, MD	Whole brain	Not Specified	WISC-IV UK	Normal	Principal Component Analysis, ANCOVA	For FAs across different tracks, the third principal component predicted full- scale IQ ($F_{1,49} = 8.36$, $P < 0.01$). For MDs across different tracks, second principal component predicted IQ ($F_{1,50} = 4.60$, $P < 0.05$)
8	Wang et al.[105]	2012	16	13-18	-	DTI	FA	Right Inferior fronto-occipital fasciculus (IFOF)	Not Specified	WAIS	Normal	TBSS	Positive correlation (value not specified) with $p = 0.05$ between FA in IFOF and FSIQ
9	Penke et al.[100]	2012	420	71-73	Lothian Birth Cohort 1936 (LBC1936)	DTI	Tract averaged FA	Whole brain	Not Specified	WAIS-III	Normal	Structural equation modelling (SEM)	SEM factor for FA explained 10% of variance in general intelligence, *g*
10	Haász et al.[107]	2013	100	49-80	Norwegian Cognitive Neurogenetics (NCNG)	DTI	FA, MD, AD, RD	Whole brain	Not Specified	California Verbal Learning Test (CVLT-II), WASI, and Color–Word Interference Test (CWIT)	Normal	TBSS	The strongest relationship between *gF* and diffusion measures was observed for FA ($r = 0.57$)
11	Dunst et al.[114]	2014	63	18-50	Locally recruited in Graz, Austria	DTI	FA, RD. AD	Whole brain	Not Specified	Intelligence-structure-battery (INSBAT)	Normal	ANOVA, Voxel-wise TBSS	There was no significant group difference in FSIQ for sex.
12	Fischer et al.[115]	2014	43	60-85	Locally recruited in University Medical Center of Mainz, Germany	DTI	FA	Whole brain	Not Specified	WAIS-revised	Normal	Partial correlation between FA and ages	Group of younger elderly showed slightly higher FSIQ than the group of advanced elderly with not statistically significance
13	Navas-Sánchez et al.[102]	2014	36	11-15	Locally recruited in Madrid, Spain	DTI	FA	Corpus Callosum	Not Specified	WISC	Normal	Pearson Correlation	FSIQ correlated with mean FA for the whole corpus callosum ($r = 0.48$; $p < 0.003$)

14	Nestor et al.[103]	2015	26	38.62±10.61	Locally recruited in Boston, MA, USA	DTI	FA	Medial orbital frontal cortex (rOFC), rostral anterior cingulate cortex (rACC)	25, 32	WAIS-III	Normal	Pearson Correlation	FA in medial orbital frontal cortex positively correlated to FSIQ ($r = 0.496$; $p = 0.01$)
15	Ohtani et al.[104]	2017	26	19-55	Locally recruited in Boston, MA, USA	DTI	FA	Medial orbital frontal cortex (rOFC), rostral anterior cingulate cortex (rACC)	25, 32	WAIS-III	Normal	Pearson Correlation	FA of the mOFC–rACC pathway is positively correlated with FSIQ ($r = 0.463$, $p = 0.020$)
16	Pisner et al.[108]	2017	32	18-45	McLean Hospital, USA	DTI	FA	Whole brain	Not Specified	Mayer–Salovey–Caruso Emotional Intelligence Test (MSCEIT)	Normal	Linear Correlation	MSCEIT Understanding Emotion positively correlated with the mean FA ($r = 0.63$; $p < 0.05$)
17	Nusbaum et al.[109]	2017	44	8-12	Lyon's Neurological Hospital, France	DTI	FA, MD, AD, RD	Whole brain	Not Specified	Child Behavior Checklist (CBCL)	Normal	TBSS	When compared to the Control group, Higher IQ groups (FSIQ > 130) presented greater AD and FA in widespread WM regions of frontal, central and associative pathways.
18	Koenis et al.[116]	2018	330	9-23	Netherland's Twin Register	DWI	FA-weighted brain networks	Whole brain	Not Specified	WAIS-III	Normal	Linear Correlation	FSIQ is found correlated to the global brain network efficiency at age 18 ($r = 0.28$; $p < 0.0001$)
19	Ponsoda et al.[117]	2017	94	20.0±1.7	Hospital Ruber Internacional, Madrid	DWI	Tractography-based brain connectivity matrix	Whole brain	Not Specified	-	Normal	Multivariate distance matrix regression (MDMR)	Individuals with similar brain connectivity profiles are also closer in their *gF* and *gC* levels
20	Kenett et al.[118]	2018	416	20.0±1.26	Southwest University, USA	DTI	Anatomical connectivity using tractography and parcellation	Inferior Parietal Lobe	42	Combined Raven's Test (CRT)	Normal	Network Control Theory	Inferior Parietal Lobe exhibited positive correlation ($r = 11\%$; $p < 0.02$) with average controllability estimated by CRT.
	Kocevar et al.[119]	2019	43	8-12	Private psychological center (PSYRENE) and the children's psychiatry unit of Lyon's Neurological Hospital, France	DTI	Tractography-based brain connectivity matrix	Whole brain	Not Specified	WISC-IV	Normal	General Linear Model	Global connectivity revealed a strong relationship between high intelligence scores and brain network homogeneity

3. Functional MRI Pre-processing and Feature Extraction

The process of acquiring and preprocessing fMRI signals (see Supplementary Figure 3) to generate a functional connectivity matrix begins with the acquisition of BOLD signals using an fMRI scanner. This signal is a measure of the change in blood oxygenation in response to neural activity. Then, a few initial volumes of the fMRI data are removed to allow the signal to reach a steady state. It usually takes around 10 seconds. Any head motion during the scan can introduce artifacts in the data. These are corrected using algorithms that align the images to a reference volume. Volume scrubbing is also performed to remove volumes with excessive motion. Then, the intensity of the fMRI signals is normalized across subjects to ensure comparability. Afterward, the fMRI data is registered to a standard brain atlas. This involves aligning the individual's brain data to a reference brain template. Noise artifacts are often contributed to the fMRI signal by the white matter and cerebrospinal fluid signals. So, these noises are usually removed from the data at this stage. The BOLD signal is typically a low-frequency signal (< 0.1 Hz). On the other hand, extremely low-frequency signals (< 0.01 Hz) are considered non-neuronal. Therefore, a bandpass filter temporally filters the physiological noise, such as cardiac and respiratory fluctuations. Afterward, the fMRI data is spatially smoothed to increase the signal-to-noise ratio and compensate for anatomical variability between

subjects. Finally, the preprocessed fMRI data is used to generate a functional connectivity matrix. This involves correlating the BOLD signal time courses between different regions of the brain.

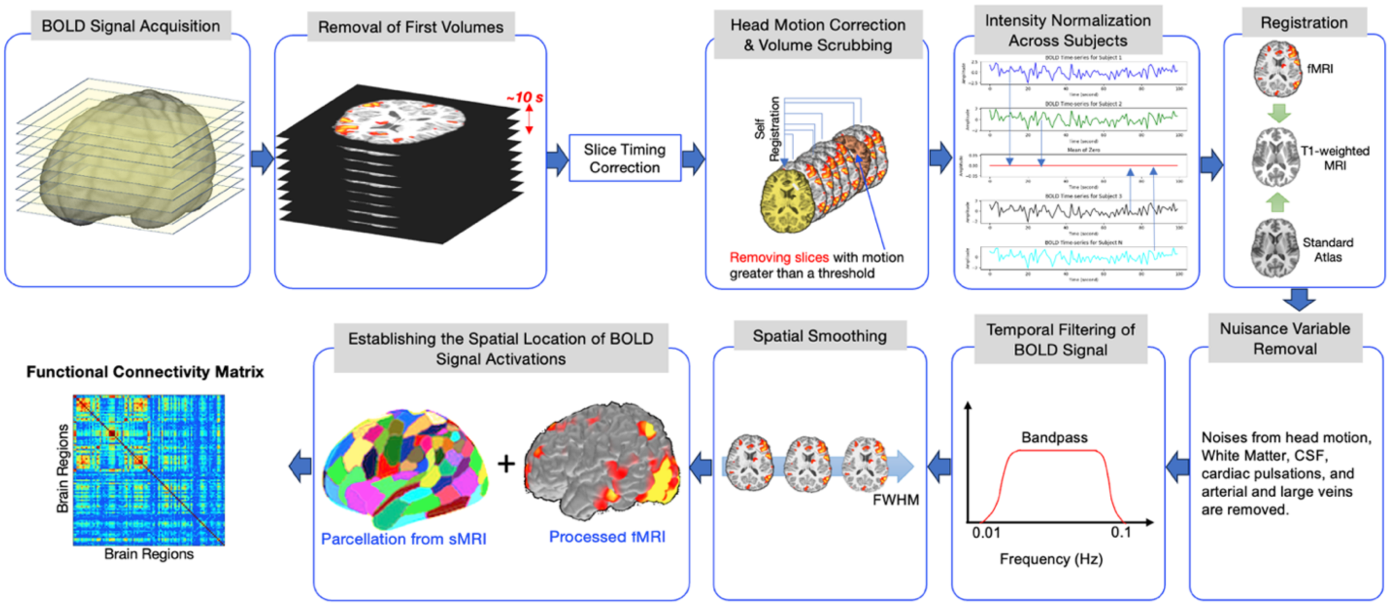

Supplementary Figure 3. *Typical functional MRI (fMRI) functional connectivity estimation pipeline.*

Supplementary Table 5. *Summary of functional MRI inferring human neurocognition and intelligence. Acronyms- OASIS: Open Access Series of Imaging Studies, KSHAP: Korean Social Life, Health, and Aging Project, ABCD: Adolescent Brain Cognitive Development, FC: Functional Connectivity, MMSE: Mini-Mental Status Examination, BOLD: Blood-oxygen-level-dependent, ABCD: Adolescent Brain Cognitive Development, NIH-TCB: NIH toolbox of neurocognitive battery, ANOVA: Analysis of Variance, FSIQ: Full-scale Intelligent Quotient, PIQ: Performance IQ, VIQ: Verbal IQ, LASSO: Least Absolute Shrinkage and Selection Operator, CNN: Convolutional Neural Network, HCP: Human Connectome Project, WAIS: Wechsler Adult Intelligence Scale, WASI: Wechsler Abbreviated Scale of Intelligence, MMSE: Mini-mental State Examination, CPM: Connectome-Based Predictive Modeling; T1-w: T1-weighted MRI, fMRI: functional MRI. Probable BAs are not specified for either the left or right hemisphere.*

Serial	Study	Year	N	Age (years)	Dataset	MRI type	MRI features	Regions	Probable BAs	IQ/Neuro. Test	Normal/ Abnormal	Method	Correlation/ Finding
1	Vakhtin et al.[123]	2014	79	21.7±3.1	Volunteers recruited in University of New Mexico (UNM), Albuquerque, NM, USA.	fMRI	Functional connectivity	Functional networks: attentional, cognitive, default-mode, sensorimotor, visual, auditory, and basal ganglia	Not Specified	Raven	Normal	Cross-correlations of the network spatial maps between resting state and Raven's sessions	Functional brain networks are found stable and maintain their general features across resting state and engagement in a complex cognitive task.
2	Schultz & Cole[124]	2016	100	-	Washington University–Minnesota Consortium Human Connectome Project	fMRI	Functional Connectivity	Whole brain	Not Specified	Raven	Normal	Similarity of FC patterns in terms of Pearson correlation of connection weights as a measure of functional network updates	High-performing individuals exhibited more efficient brain connectivity updates in the form of smaller changes in functional network architecture between rest and task.
3	Kruschwitz et al.[125]	2018	1,096	28.8±3.7	HCP	fMRI	Whole-brain voxel-level networks	Whole brain	Not Specified	NIH-TCB	Normal	Partial correlation	This study did not observe any significant

												associations to characteristic path length and global efficiency in any of the network parcellation schemes.
4	Pezoulas et al.[126]	2017	136	22~36	HCP	fMRI	Functional Connectivity	Cerebellum	Not Specified	Raven	Normal	ANOVA	high-IQ females have higher average clustering coefficient than high-IQ males (high-IQ males: 1.15 ± 0.039; high-IQ females: 1.17 ± 0.065), as well as characteristic path-length (high-IQ males: 0.94±0.087; high-IQ females: 0.97 ± 0.046).
5	Noble et al.[127]	2017	618	22-56	HCP	fMRI	10 functionally coherent networks	Whole gray matter	Not Specified	Raven's Progressive Matrices	Normal	CPM	Correlation between actual and estimated *gF* is 22% ($p < 0.0001$)
6	Wang et al.[129]	2011	59	18.5-33.3	Local Hospital	rs-fMRI	Regional homogeneity (ReHo) of local connectivity	Right middle frontal gyrus, left middle frontal gyrus, right superior frontal gyrus, right inferior parietal lobule, left inferior parietal lobule, left superior parietal lobule, parahippocampal gyrus, right inferior temporal gyrus, left inferior temporal gyrus, right fusiform gyrus, right thalamus	6, 8, 9, 10, 46, 47, 7, 39, 40, 20, 21, 28, 30, 34, 37	WAIS-RC	Normal	Voxel-wise partial correlation	FSIQ scores were positively correlated with the ReHo of the BA areas mentioned.
7	Langeslag et al.[130]	2013	115	5.4-7	Erasmus Medical Center, Rotterdam	rs-fMRI	Functional connectivity	Right parietal region,<br>Right frontal region, right parietal region,<br>Dorsal anterior cingulate cortex (ACC)	40, 9, 10, 46, 32	Snijders-Oomen Nietverbale intelligent Test	Normal	Hierarchical regression analyses	A significant, positive association ($\beta = 0.27\sim0.31$; $p < 0.01$) was observed between IQ and functional connectivity between the right parietal region and the right frontal region and the dorsal ACC.
8	Basten et al.[131]	2013	52	19-27	Students at the University of Heidelberg, Germany	fMRI	BOLD signal change	Anterior medial frontal cortex, superior frontal<br>gyrus, superior temporal gyrus, posterior cingulate cortex, precuneus<br>Inferior frontal sulcus, middle and inferior frontal gyri<br>Inferior frontal junction area, superior temporal gyrus, middle temporal gyrus, posterior middle temporal gyrus, hippocampus and<br>cerebellum	8, 9, 10, 11, 12, 13, 22, 24, 32, 41, 42, 43, 23, 31, 46, 6, 8, 44, 13, 22, 41, 42, 37, 21, 39, 37, 27	Raven	Normal	*t*-statistics	IQ scores were positively correlated with the BOLD signal in the mentioned regions ($T_{max} = 4.04\sim6.53$).
9	Pamplona et al.[132]	2015	29	26.8±5.8	Local volunteers from the state of São Paulo, Brazil.	fMRI	Functional connectivity	Frontal, pre-central, parietal, occipital, fusiform and supramarginal<br>gyrus, and caudate nucleus.	37, 7	WAIS-III	Normal	Pearson correlation	Functional connectivity between right fusiform and left superior parietal lobe shows a correlation of $r=0.62$ (FDR = 0.003) with FSIQ.
10	Hilger et al.[133]	2017	54	18-30	Nathan S. Kline Institute (NKI) for Psychiatric Research, NY	fMRI	Individual brain networks as unweighted thresholded graph	Whole brain, however, prominently right anterior insula (AI) and dorsal anterior cingulate cortex (dACC).	13, 47, 32	WASI	Normal	Global graph theoretical measure of graph efficiency	A positive association between intelligence and nodal efficiency is found in right AI and dACC (AI: $t_{max}=3.38$; dACC: $t_{max}=3.89$).

11	Hilger et al.[134]	2017	309	18-60	Nathan S. Kline Institute (NKI) for Psychiatric Research, NY	fMRI	Thresholded and binarized graphs constructed from BOLD activation	Whole brain, however, prominently right anterior insula, superior frontal gyrus (SFG), and temporo-parietal junction (TPJ)	13, 47, 9, 10, 39, 40	WASI	Normal	Global graph theoretical measure of graph efficiency	In higher FSIQ population, right AI showed higher connectivity to other modules In contrast, medial SFG and bilateral TPJ showed higher connectivity within-module.
12	Hearne et al.[135]	2016	317	22-36	HCP	fMRI	Functional Connectivity	Whole brain	Not Specified	Penn's Progressive Matrices (PMAT) for *gF* and Picture Vocabulary Test from NIH-TCB for *gC*	Normal	Network based statistic	Significant patterns of pairwise functional connectivity between prefrontal and frontal cortices positively associated with intelligence (r=0.38)
13	Santarnecchi et al.[136]	2017	130	18-55	NKI-Rockland	fMRI	Resting state network and *gF* activation pattern	Whole brain, however, prominently inferior frontal gyrus and inferior parietal lobule	6, 40	Raven	Normal	Spatial similarity	*gF* networks have positive correlated structures to two major hubs, located in the inferior frontal gyrus [F (1130) = 6.246, p < 0.05] and inferior parietal lobule [F (1130) = 5.145, p < 0.05]
14	Kwak et al.[148]	2021	795	46-96	OASIS-3, KSHAP	T1-w, fMRI	Functional connectivity from BOLD signals	Region of frontoparietal network and central brain	9, 4, 39, 40, 46, 10, 13, 1, 2, 3	MMSE	Normal	Ridge regression	Correlation between behavioral test scores and FC-predicted score is 0.12~0.44 (p < 0.001).
15	Finn et al.[149]	2015	126	22-35	Human Connectome Project (HCP)	fMRI	Positive and negative edges, frontoparietal networks	Frontoparietal region	9, 4, 39, 40, 46, 10, 13	Raven's Progressive Matrices	Normal	Connectome-Based Predictive Modeling (CPM)	Correlation between actual and estimated *gF* is 0.5 (p < 0.01)
16	Powell et al.[150]	2017	841	22-37	HCP	fMRI	Voxel-wise local structural connectome	Region of frontoparietal network	9, 4, 39, 40, 46, 10, 13	NIH-TCB	Normal	LASSO Principal Component Regressor	Correlation between the actual and predicted NIH picture sequence memory test is 0.097 (p < 0.001)
17	Sripada et al.[151]	2020	2,013	9-10	ABCD	fMRI	Resting-state functional connectivity pattern	Default mode network, frontoparietal network, salience network, dorsal attention network	8, 9, 10, 21, 28, 36, 23, 24, 32, 29, 30, 31, 39, 40	NIH-TCB	Normal	Brain basis set (BBS) modeling (combination of PCA and linear regression)	General neurocognitive ability score is highly correlated to the mentioned networks (r = 0.31; p < 0.0001)
18	Jiang et al.[152]	2017	360	17-24	University of Electronic Science and Technology, China	fMRI	Functional connectivity	Superior frontal gyrus, inferior and superior parietal lobules	10, 11, 12, 39, 40, 7	WAIS-RC	Normal	RelieF+LASSO	Correlation between actual and estimated FSIQ is 51% (p < 0.001)
19	Ebisch et al.[137]	2012	22	20-24	Local female student volunteers	fMRI	Functional connectivity	bilateral superior frontal gyrus/sulcus, inferior parietal lobe, posterior parietal cortex, superior parietal cortex, anterior insular cortex, temporal–occipital cortex, dorsolateral prefrontal cortex, precentral gyrus, anterior cingulate cortex, superior parietal cortex, dorsal extrastriate cortex, thalamus, and cerebellum.	40, 11, 12, 7, 13, 14, 16, 4, 24, 32, 33	Fluid intelligence test (FIT)	Normal	Group statistical map	*gF* tasks activate a shared frontoparietal network.
20	Cole et al.[138]	2012	121	18-40	Undergraduate student volunteers in the Washington University.	fMRI	Global brain connectivity (GBC)	Lateral prefrontal cortex (LPFC)	9, 10, 46	Raven	Normal	Linear regression	*gF* is found correlated to GBC in LPFC region (r = 0.32; p = 0.0015)

21	Cole et al.[139]	2015	121	18-40	Undergraduate student volunteers in the Washington University.	fMRI	Global brain connectivity (GBC)	Lateral prefrontal cortex (LPFC)	9, 10, 46	Raven	Normal	Linear regression	*gF* is found correlated to GBC in LPFC region (r = 0.28; p = 0.006)
22	Greene et al.[141]	2018	1,086	8-36	HCP, Philadelphia Neurodevelopmental Cohort (PNC)	fMRI	Whole brain functional connectivity	Cortical and subcortical grey matter, cerebellum	Not Specified	Raven's Progressive Matrices	Normal	CPM	Correlation between actual and estimated *gF* is 19% in resting state (p = 0.039)
23	He et al.[143]	2018	9,821	22-69	HCP, UK-Biobank	fMRI	Functional Connectivity Matrix	Whole-brain spatially independent components	Not Specified	Raven's Progressive Matrices	Normal	Kernel Regression, Feedforward NN, CNN	Correlation between actual and estimated *gF* is 23.9% (p < 0.001) using the Kernel regression
24	Li et al.[144]	2018	100	-	HCP	fMRI	Amplitude of low-frequency fluctuation of left anterior cingulate cortex	Right prefrontal cortex, left anterior cingulate cortex	8, 24, 32, 33	Raven's Progressive Matrices	Normal	Support vector regressor	Correlation between actual and estimated *gF* is 32.5% (p = 0.031)
25	Dubois et al.[145]	2018	884	22-36	HCP	fMRI	Functional Connectivity Matrix	Cortical and subcortical grey matter	Not Specified	Raven's Progressive Matrices	Normal	Univariate correlation filtering + Elastic net regression	Correlation between actual and estimated *gF* is 22% using the univariate model (p < 0.001)
26	Yoo et al.[146]	2019	575	22-56	HCP	fMRI	Functional Connectivity Matrix	Regions of frontoparietal and default mode networks	9, 4, 39, 40, 46, 10, 13, 38, 25, 23, 31	Raven's Progressive Matrices	Normal	CPM-based Multivariate distance correlation	Correlation between actual and estimated cognitive ability is 9.5% (p < 0.01)
27	Graham et al.[147]	2010	28	Average IQ Group: 29.9 ± 11.9; High IQ Group: 26.3 ± 12.2	Local Hospital	fMRI	BOLD activation	Whole brain	3, 4, 6, 7, 8, 9, 19, 31, 32, 38, 46, 47	WASI	Normal	Change of signals across events	Greater BOLD activation across different brain regions (e.g., parietal, caudate, fusiform and occipital) is seen for complex reasoning in high-IQ group.

4. Deep Learning of Brain MRI and Neurocognition

Supplementary Table 6. *Summary of MRI studies inferring/relating to human neurocognition and intelligence Using Deep Learning. Acronyms- ANCOVA: Analysis of Covariance, ABCD: Adolescent Brain Cognitive Development, NIH-TCB: NIH toolbox of neurocognitive battery, LASSO: Least Absolute Shrinkage and Selection Operator, SVM: Support Vector Machine, SVR: Support Vector Regression, CNN: Convolutional Neural Network, ROI: Region of Interest, KNN: K-Nearest Neighbors, WASI: Weschler Abbreviated Scale of Intelligence, WISC: Wechsler Intelligence Scale for Children, WAIS: Wechsler Adult Intelligence Scale, FSIQ: Full-scale Intelligent Quotient, ABIDE: Autism Brain Imaging Data Exchange, T1-w: T1-weighted MRI, T2-w: T2-weighted MRI, DWI: Diffusion-weighted Imaging, DTI: Diffusion Tensor Imaging, TRUST: T2-relaxation under spin tagging. Probable BAs are not specified for either the left or right hemisphere.*

**Serial**	**Study**	**Year**	**N**	**Age (years)**	**Dataset**	**MRI type**	**MRI features**	**Regions**	**Probable BAs**	**IQ/Neuro. Test**	**Normal/ Abnormal**	**Method**	**Correlation/ Finding**
1	Saha et al.[63]	2021	7,709	9-10	ABCD	T1-w	CNN learned features and volumes of manually identified brain regions	GM regions of left/right hippocampus, parahippocampal gyrus, thalamus, precentral gyrus and caudate nucleus; WM region of the pons.	34, 4	NIH-TCB	Normal	CNN and MLP	Correlation between the actual and predicted *gF* = 0.1 (p < 0.05)
2	Chiang et al.[53]	2019	8,669	9-10	ABCD	T1-w	Total volume, mean signal intensity, and entropy	Visual, frontoparietal, somatosensory, motor, default mode network, and cingulo-opercular network.	6, 8, 9, 22, 41, 42, 17, 18, 19, 1, 2, 3, 5, 7, 4	NIH-TCB	Normal	CNN, and LASSO	Mean Square Error *(gF)* = 95.38 (for true residual *gF* in the range of [-40, 30])
3	Ranjbar et al.[59]	2019	8,669	9-10	ABCD	T1-w	122 ROI volumes in the gray matter, white matter, and cerebrospinal fluid	Gray matter, white matter, and cerebrospinal fluid	11, 44, 45, 47, 4, 1, 2, 3, 10, 12, 40	NIH-TCB	Normal	CNN and random forest	Mean Square Error *(gF)* = 93.64 (for true residual *gF* in the range of [-40, 30])
4	Vang et al.[153]	2019	8,669	9-10	ABCD	T1-w	CNN-learned features	Gray matter, white matter, and cerebrospinal fluid	Not Specified	NIH-TCB	Normal	CNN with gradient boosting machine	Mean Square Error *(gF)* = 96.18 (for true residual *gF* in the range of [-40, 30])
5	Pominova et al.[154]	2019	8,669	9-10	ABCD	T1-w	CNN-learned features	Gray matter	Not Specified	NIH-TCB	Normal	VoxCNN	Mean Square Error *(gF)* = 93.838 (for true residual *gF* in the range of [-40, 30])

6	Zou et al.[155]	2019	8,669	9-10	ABCD	T1-w	CNN-learned features	Bilateral transverse temporal gyri, bilateral thalamus, left parahippocampal gyrus, left hippocampus, right opercular part of inferior frontal gyrus, left anterior cingulate gyrus, right amygdala, left lingual gyrus, left superior parietal lobule, right inferior parietal lobule, left angular gyrus, left paracentral lobule, and left caudate nucleus.	41, 42, 34, 44, 45, 47, 24, 32, 33, 19, 7, 39, 40, 1, 2, 3, 4	NIH-TCB	Normal	3D CNN	Mean Square Error *(gF)* = 92.74 (for true residual *gF* in the range of [-40, 30])
7	Liu et al.[156]	2019	8,669	9-10	ABCD	T1-w	CNN-learned features	Skull-stripped whole brain	Not specified	NIH-TCB	Normal	UNet-like encoder/decoder	Mean Square Error *(gF)* = 102.25 (for true residual *gF* in the range of [-40, 30])
